# Association Between Handgrip Strength and Cardiovascular Disease Risk in MASLD: A Prospective Study From UK Biobank

**DOI:** 10.1002/jcsm.13757

**Published:** 2025-03-04

**Authors:** Tae Seop Lim, Sujin Kwon, Sung A. Bae, Hye Yeon Chon, Seol A. Jang, Ja Kyung Kim, Chul Sik Kim, Seok Won Park, Kyoung Min Kim

**Affiliations:** ^1^ Department of Internal Medicine Yonsei University College of Medicine Seoul Republic of Korea; ^2^ Department of Gastroenterology, Internal Medicine Yongin Severance Hospital Yonsei University Health System Yongin Republic of Korea; ^3^ Department of Endocrinology, Internal Medicine Yongin Severance Hospital Yonsei University Health System Yongin Republic of Korea; ^4^ Department of Cardiology, Internal Medicine Yongin Severance Hospital Yonsei University Health System Yongin Republic of Korea

**Keywords:** cardiovascular disease, fatty liver, handgrip strength, metabolic dysfunction‐associated steatotic liver disease, muscle strength

## Abstract

**Background:**

This study aimed to investigate the association between handgrip strength (HGS) and cardiovascular disease (CVD) in individuals with metabolic dysfunction‐associated steatotic liver disease (MASLD) using data from the UK Biobank cohort.

**Methods:**

A total of 201 563 participants were enrolled in this study. The HGS was measured using a Jamar J00105 hydraulic hand dynamometer. MASLD was defined as the presence of hepatic steatosis accompanied by one or more cardiometabolic criteria. Hepatic steatosis was identified using a fatty liver index ≥ 60. Advanced liver fibrosis was defined by a fibrosis‐4 (FIB‐4) score > 2.67. To examine the differences in the incidence of CVD, male and female participants were divided into non‐MASLD, MASLD with high HGS, MASLD with middle HGS, and MASLD with low‐HGS groups.

**Results:**

Of the study participants, 75 498 (37.5%) were diagnosed with MASLD, with a mean age of 56.5 years, and 40.6% were male. The median follow‐up duration was 13.1 years. The frequency of incident CVD events increased significantly across groups: 10.9% in non‐MASLD, 13.3% in MASLD with high HGS, 14.8% in MASLD with middle HGS, and 18.4% in MASLD with low HGS for males (*p* < 0.001). In females, the frequency of incident CVD events was 6.1% in non‐MASLD, 9.2% in MASLD with high HGS, 10.7% in MASLD with middle HGS, and 13.3% in MASLD with low HGS (*p* < 0.001). Using the non‐MASLD group as a reference, multivariate‐adjusted hazard ratios (HRs) (95% confidence intervals [CI]) for CVD varied according to HGS in individuals with MASLD. In males with MASLD, HRs (95% CI) were 1.03 (0.96–1.10) for high HGS, 1.14 (1.07–1.21) for middle HGS, and 1.38 (1.30–1.46) for low HGS; in females with MASLD, they were 1.07 (0.97–1.18) for high HGS, 1.25 (1.14–1.37) for middle HGS, and 1.56 (1.43–1.72) for low HGS. The incidence of CVD events increased as HGS decreased in participants with MASLD, regardless of the presence or absence of advanced liver fibrosis (all *p* < 0.001).

**Conclusions:**

This large prospective cohort study using the UK Biobank showed that in MASLD, a decrease in HGS was associated with increased CVD risk.

## 1 Introduction

Metabolic dysfunction‐associated steatotic liver disease (MASLD), previously referred as non‐alcoholic fatty liver disease (NAFLD), has a rapidly increasing prevalence, now affecting nearly one in three individuals worldwide [1]. This surge is expected to persist in the forthcoming years, propelled by escalating obesity rates, an aging demographic, unhealthy diet, and the widespread adoption of sedentary lifestyles [2]. MASLD, while inherently posing a risk of progressing to more severe liver conditions such as metabolic dysfunction‐associated steatohepatitis, liver cirrhosis, and hepatocellular carcinoma, also extends its influence beyond the liver, increasing the risk of cardiovascular diseases (CVD) and extrahepatic cancers, among other complications [3].

Emerging research underscores a robust association between MASLD and an elevated CVD risk [4, 5]. MASLD serves as an independent risk factor for CVD, comparable to other well‐recognized risk factors such as hypertension, dyslipidaemia, and diabetes mellitus; most importantly, CVD‐related mortality is the leading cause of death among those affected by MASLD [6]. This underscores the relationship between MASLD and cardiovascular complications.

Identifying high‐risk groups among individuals with MASLD for potential onset of CVD is crucial. However, reliance on conventional metrics such as smoking, hypertension, dyslipidaemia, and diabetes mellitus might not paint a complete picture [7]. Muscle tissue, a vital metabolic organ, plays a crucial role in regulating glucose and lipid metabolism [8]. Moreover, enhanced muscle strength is positively correlated with improved cardiac function [9]. Thus, recent findings indicate that decreased muscle strength could emerge as a significant indicator of CVD risk [10, 11, 12]. However, the longitudinal studies examining whether and how much the CVD risk in MASLD varies according to muscle strength are scarce.

Considering the existing knowledge gap, this study used extensive data from the UK biobank database to explore how the risk of incident CVD varies among individuals with MASLD based on muscle strength as measured by handgrip strength (HGS).

## 2 Methods

### 2.1 Data Source

The UK Biobank is an extensive prospective cohort based on the population. More than half a million men and women visited one of the 22 centres throughout the UK from 2006 to 2010 [13]. The UK Biobank received ethical clearance from the North West Multi‐Centre Research Ethics Committee (16/NW/0274). The research was carried out under UK Biobank application 85 037. The study was conducted in accordance with the Declaration of Helsinki and Istanbul guidelines, and every participant provided written informed consent before participation. Each participant completed a questionnaire on a touchscreen device, underwent physical assessments, and provided blood, urine, and saliva samples when they joined the study. Further details of the UK Biobank protocol are available on its official website (http://www.ukbiobank.ac.uk).

### 2.2 Study Population

**FIGURE 1:**
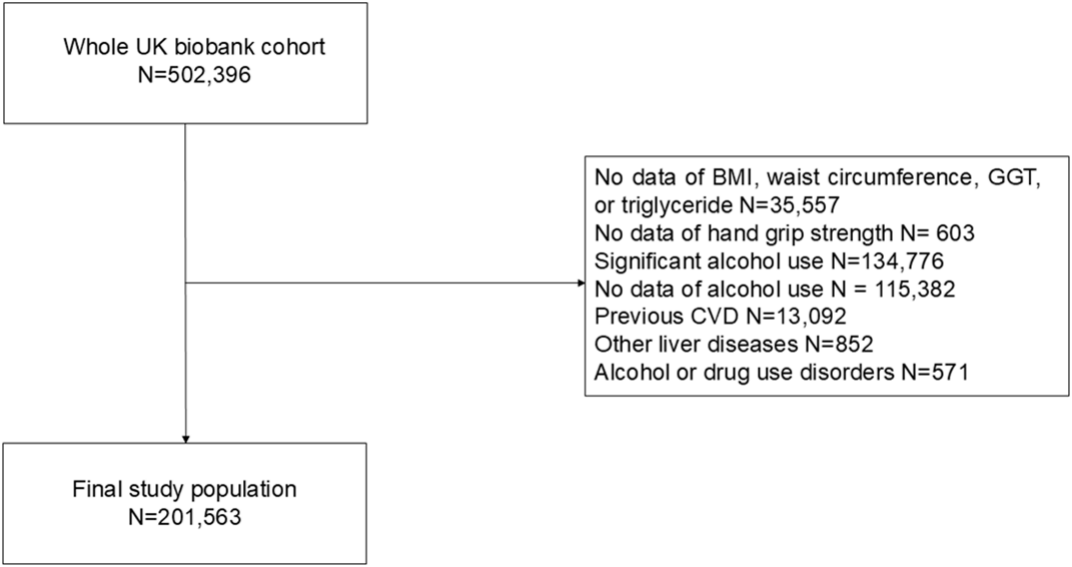
Study flow chart. Abbreviations: BMI, body mass index; CVD, cardiovascular disease; GGT, gamma‐glutamyl transferase.

We identified 502 396 adults who participated in the UK Biobank Cohort Study between 2006 and 2010. The follow‐up period began on the day of the baseline examination, which was also the index date when HGS was measured. After excluding participants with missing fatty liver index (FLI) variables (*n* = 35 557), missing HGS data (*n* = 603), significant alcohol use (*n* = 134 776), missing alcohol use data (*n* = 115 382), previous CVD (*n* = 13 092), other liver diseases (*n* = 852), or alcohol or drug use disorders (*n* = 571), a final analytical population of 201 563 participants was included (Figure 1). Significant alcohol use was defined as > 210 g per week for men and > 140 g per week for women [1, 14, 15]. Individuals diagnosed with other liver diseases or alcohol or drug‐use disorders were defined based on International Classification of Diseases (ICD)‐10 codes (Table S1) [16]. Past disease at baseline was defined as self‐reported or physician diagnosis (Table S2).

### 2.3 MASLD and Advanced Liver Fibrosis

MASLD was defined as the presence of hepatic steatosis accompanied by one or more cardiometabolic criteria: (1) body mass index (BMI) ≥ 25 kg/m^2^ (23 in Asian) or waist circumference > 94 cm (for males) or 80 cm (for females); (2) fasting serum glucose ≥100 mg/dL or type 2 diabetes or treatment for type 2 diabetes; (3) blood pressure ≥ 130/85 mmHg or specific antihypertensive drug treatment; (4) triglycerides ≥ 150 mg/dL or lipid‐lowering medication; (5) and high‐density lipoprotein (HDL) cholesterol < 40 mg/dL for males and < 50 mg/dL for females or lipid‐lowering medication [1, 14, 15].

According to other epidemiological studies, hepatic steatosis identification was based on FLI ≥ 60 [17, 18, 19]. In extensive epidemiological research, the European Clinical Practice Guidelines recognize FLI as a viable substitute for imaging techniques [20]. Advanced liver fibrosis was defined by a fibrosis‐4 score (FIB‐4) > 2.67 [17, 21]. The definition of hepatic steatosis and advanced liver fibrosis was described in Table S3.

### 2.4 Handgrip Strength

HGS assessment was conducted using a Jamar J00105 hydraulic hand dynamometer. We used only the highest measurements recorded from each hand [10]. HGS varied significantly based on sex and age. Thus, we first divided the participants into groups based on age within each sex: < 50 years, 50–60 years, 60–70 years, and > 70 years. Then, the z‐scores for HGS for the participants were calculated based on the mean and standard deviations of each age group [22, 23]. Subsequently, HGS was categorized as follows: low HGS with a z‐score below −0.5, middle HGS with a z‐score between −0.5 and 0.5, and high HGS with a z‐score > 0.5.

### 2.5 Outcomes

Previous and incident CVD events in the present study were defined using two main diagnostic approaches. The first diagnostic approach was through ICD‐10 codes, where CVD encompasses acute myocardial infarction (I21), subsequent myocardial infarction (I22), certain current complications following acute myocardial infarction (I23), angina pectoris (I20), other acute ischemic heart diseases (I24), chronic ischemic heart disease (I25), and stroke not specified as haemorrhage or infarction (I64) [24]. Second, CVD is defined algorithmically via specific fields capturing the date of myocardial infarction (Field 42000), the date of STEMI (Field 42002), the date of NSTEMI (Field 42004), the date of stroke (Field 42006), and the date of ischemic stroke (Field 42008) [24].

### 2.6 Statistical Analysis

Baseline characteristics are presented as frequency, percentage, or mean values with the corresponding standard deviations. The study participants were stratified into two groups based on the presence of MASLD, which were further stratified into three groups according to HGS: non‐MASLD, MASLD with high HGS, MASLD with middle HGS, and MASLD with low HGS. The incidence of CVD events based on HGS was compared using the chi‐square test. The cumulative incidence of CVD events was analysed using the Kaplan–Meier approach. Hazard ratios (HRs) and 95% confidence intervals (CIs) were determined using a Cox proportional hazards model. The HRs were adjusted for BMI, smoking status, diabetes mellitus, hypertension, dyslipidaemia, and physical activity. Physical activity was presented as the metabolic equivalent of task minutes per week.

Participants with missing values for variables essential to the primary analysis were excluded according to predefined exclusion criteria (Figure 1). Specifically, we excluded participants missing values for any component of the FLI, including BMI, waist circumference, gamma‐glutamyl transferase, or triglyceride (*n* = 35 557), as well as those missing HGS data (*n* = 603). For other variables, we retained missing values as ‘not available’ due to minimal missing rates; most variables had a completion rate over 97%, with only four variables (albumin, glucose, haemoglobin A1c, and HDL cholesterol) having completion rates between 90% and 94%. Physical activity data, collected via self‐reported touchscreen responses in the UK Biobank, had a completion rate of 84% for male participants and 77% for female participants, with missing values for 12 992 males and 27 348 females. Given that physical activity (measured as metabolic equivalent of task (MET) minutes per week) was not a primary focus of our analysis, we retained these missing values without imputation to prevent potential bias and maintain dataset size. In addition, to address baseline differences between the non‐MASLD and MASLD groups and to achieve a balanced comparison, sensitivity analyses were conducted, and an exact propensity score matching (PSM) approach was employed. In the PSM approach, participants from the non‐MASLD and MASLD groups were matched based on key categorical variables, including age (categorized), ethnicity, smoking status, diabetes mellitus, hypertension, and dyslipidaemia. Analyses were conducted using the R version 4.1.2 (R Foundation for Statistical Computing, Vienna, Austria).

## 3 Results

### 3.1 Baseline Characteristics

The baseline characteristics of the study participants, differentiated by sex and the presence of MASLD, are demonstrated in Table 1. The study finally enrolled 201 563 participants with a mean age of 56.5 years and 40.6% being male. Among these, 75 498 (37.5%) were diagnosed with MASLD. The median follow‐up duration was 13.1 years. Participants with MASLD were generally older than those with non‐MASLD. They also exhibit a higher prevalence of diabetes mellitus, hypertension, and dyslipidaemia. These individuals had an increased BMI and waist circumference. Regarding lifestyle factors, the participants with MASLD were less engaged in physical activities. Clinically, these participants demonstrate higher systolic and diastolic blood pressures, elevated platelet counts, and increased aspartate aminotransferase, alanine aminotransferase, gamma‐glutamyl transferase, creatinine, and glucose levels. Haemoglobin A1c levels were also elevated. Regarding lipid profiles, the participants with MASLD had higher levels of total cholesterol, low‐density lipoprotein (LDL) cholesterol, triglycerides, and lower HDL cholesterol levels. Baseline characteristics according to HGS are described in Table S4. As noted, participants in the high, middle, and low HGS groups exhibited distinct characteristics. Among males, those in the middle and low HGS groups were more likely to be current smokers, and had a higher prevalence of diabetes mellitus, hypertension, and dyslipidaemia compared to those in the high HGS group. Additionally, participants in the middle and low HGS groups had lower BMI values and a more sedentary lifestyle. Similar patterns were observed among females.

**TABLE 1:** Baseline characteristics.

Variables	Male				Female			
	Overall	Non‐MASLD	MASLD	*p*	Overall	Non‐MASLD	MASLD	*p*
*n*	81 915	39 670	42 245		119 648	86 395	33 253	
Age, years	56.5 ± 8.3	56.2 ± 8.5	56.7 ± 8.2	< 0.001	56.6 ± 8.1	56.1 ± 8.2	57.7 ± 7.6	< 0.001
Age (%)				< 0.001				< 0.001
Under 50	20 294 (24.8)	10 470 (26.4)	9825 (23.3)		27 683 (23.1)	21 889 (25.3)	5794 (17.4)	
50–60	25 336 (30.9)	11 869 (29.9)	13 469 (31.9)		39 771 (33.2)	28 514 (33.0)	11 257 (33.9)	
60–70	35 846 (43.8)	17 091 (43.1)	18 756 (44.4)		51 644 (43.2)	35 609 (41.2)	16 035 (48.2)	
Over 70	439 (0.5)	240 (0.6)	199 (0.5)		550 (0.5)	383 (0.4)	167 (0.5)	
Race				< 0.001				< 0.001
White	73 820 (90.1)	35 498 (89.5)	38 322 (90.7)		108 855 (91.0)	78 850 (91.3)	30 005 (90.2)	
Black	2189 (2.7)	1216 (3.1)	973 (2.3)		3231 (2.7)	2007 (2.3)	1224 (3.7)	
Asian	3798 (4.6)	1942 (4.9)	1856 (4.4)		4321 (3.6)	3214 (3.7)	1107 (3.3)	
Others	2108 (2.6)	1014 (2.6)	1094 (2.6)		3241 (2.7)	2324 (2.7)	917 (2.8)	
Smoking (%)				< 0.001				< 0.001
Never	42 327 (51.9)	22 520 (57.0)	19 809 (47.1)		74 164 (62.2)	54 488 (63.3)	19 676 (59.5)	
Previous	29 171 (35.8)	12 247 (31.0)	16 925 (40.2)		34 483 (28.9)	24 134 (28.0)	10 349 (31.3)	
Current	10 081 (12.4)	4766 (12.1)	5316 (12.6)		10 547 (8.8)	7493 (8.7)	3054 (9.2)	
Diabetes mellitus (%)	5920 (7.2)	1612 (4.1)	4308 (10.2)	< 0.001	5069 (4.2)	1680 (1.9)	3389 (10.2)	< 0.001
Hypertension (%)	24 101 (29.4)	8094 (20.4)	16 007 (37.9)	< 0.001	28 865 (24.1)	15 372 (17.8)	13 493 (40.6)	< 0.001
Dyslipidaemia (%)	15 669 (19.1)	5330 (13.4)	10 338 (24.5)	< 0.001	14 906 (12.5)	7676 (8.9)	7230 (21.7)	< 0.001
Body mass index, kg/m^2^	27.8 ± 4.3	24.9 ± 2.4	30.5 ± 3.9	< 0.001	27.4 ± 5.4	25.0 ± 3.2	33.7 ± 5.0	< 0.001
Waist circumference, cm	96.7 ± 11.6	88.7 ± 6.9	104.1 ± 9.9	< 0.001	85.4 ± 13.0	79.5 ± 8.3	100.7 ± 10.2	< 0.001
Physical activity (%)^a^				< 0.001				< 0.001
< 600	12 729 (15.5)	4829 (12.2)	7900 (18.7)		16 802 (14.0)	10 562 (12.2)	6240 (18.8)	
600–3000	33 199 (40.5)	16 380 (41.3)	16 819 (39.8)		46 825 (39.1)	34 940 (40.4)	11 885 (35.7)	
≥ 3000	22 995 (28.1)	12 651 (31.9)	10 344 (24.5)		28 673 (24.0)	22 726 (26.3)	5947 (17.9)	
Systolic blood pressure, mmHg	142.7 ± 18.6	139.8 ± 18.6	145.5 ± 18.1	< 0.001	137.9 ± 20.5	135.7 ± 20.5	143.6 ± 19.4	< 0.001
Diastolic blood pressure, mmHg	84.1 ± 10.5	81.6 ± 10.2	86.4 ± 10.2	< 0.001	80.8 ± 10.6	79.2 ± 10.3	85.0 ± 10.1	< 0.001
Handgrip strength, kg	40.8 ± 9.0	40.6 ± 8.7	40.9 ± 9.2	< 0.001	24.4 ± 6.4	24.7 ± 6.3	23.8 ± 6.7	< 0.001
Handgrip strength (%)				0.125				< 0.001
High	24 250 (29.6)	11 610 (29.3)	12 640 (29.9)		39 366 (32.9)	28 603 (33.1)	10 763 (32.4)	
Middle	32 728 (40.0)	15 913 (40.1)	16 815 (39.8)		47 194 (39.4)	34 381 (39.8)	12 813 (38.5)	
Low	24 937 (30.4)	12 147 (30.6)	12 790 (30.3)		33 088 (27.7)	23 411 (27.1)	9677 (29.1)	
Platelet count, 10^9^/L	231.8 ± 54.7	230.8 ± 55.0	232.8 ± 54.4	< 0.001	261.3 ± 60.0	257.1 ± 58.5	272.1 ± 62.3	< 0.001
Aspartate aminotransferase, U/L	28.0 ± 10.8	26.2 ± 8.6	29.6 ± 12.3	< 0.001	24.6 ± 9.4	23.8 ± 7.4	26.6 ± 13.1	< 0.001
Alanine aminotransferase, U/L	26.9 ± 14.9	21.8 ± 9.8	31.7 ± 17.1	< 0.001	20.2 ± 12.0	18.0 ± 9.1	25.7 ± 16.1	< 0.001
Albumin, g/dL	4.6 ± 0.3	4.6 ± 0.3	4.6 ± 0.3	0.056	4.5 ± 0.3	4.5 ± 0.3	4.4 ± 0.3	< 0.001
Total bilirubin, mg/dL	0.6 ± 0.3	0.6 ± 0.3	0.6 ± 0.3		0.5 ± 0.2	0.5 ± 0.2	0.4 ± 0.2	< 0.001
Gamma glutamyl transferase, U/L	42.9 ± 43.8	29.2 ± 19.3	55.7 ± 55.0	< 0.001	29.7 ± 31.6	23.3 ± 17.2	46.1 ± 49.5	< 0.001
Creatinine, mg/dL	0.82 ± 0.19	0.81 ± 0.18	0.83 ± 0.21	< 0.001	0.65 ± 0.15	0.64 ± 0.14	0.66 ± 0.16	< 0.001
Glucose, mg/dL	94.1 ± 24.3	91.3 ± 18.5	96.6 ± 28.5	< 0.001	92.2 ± 19.6	90.3 ± 14.9	97.2 ± 27.6	< 0.001
Haemoglobin A1c, %	5.5 ± 0.7	5.4 ± 0.5	5.6 ± 0.8	< 0.001	5.5 ± 0.6	5.4 ± 0.4	5.7 ± 0.8	< 0.001
Total cholesterol, mg/dL	212.8 ± 42.7	209.3 ± 39.6	216.2 ± 45.2	< 0.001	227.3 ± 43.8	226.2 ± 42.1	230.2 ± 47.6	< 0.001
HDL‐cholesterol, mg/dL	49.2 ± 12.0	53.2 ± 12.3	45.4 ± 10.3	< 0.001	60.5 ± 14.5	63.6 ± 14.3	52.6 ± 11.5	< 0.001
LDL‐cholesterol, mg/dL	135.6 ± 32.7	132.7 ± 30.9	138.4 ± 34.1	< 0.001	141.0 ± 33.7	139.0 ± 32.5	146.2 ± 36.1	< 0.001
Triglycerides, mg/dL	173.5 ± 101.0	123.8 ± 55.9	220.1 ± 111.2	< 0.001	140.8 ± 77.7	118.9 ± 55.8	197.7 ± 95.8	< 0.001

*Note:* Values are shown as frequency (%) or mean ± standard deviation.

Abbreviations: HDL, high‐density lipoprotein; LDL, low‐density lipoprotein; MASLD, metabolic dysfunction‐associated steatotic liver disease.

^a^ Physical activity is presented as a metabolic equivalent of task minutes per week. There are 12 992 missing data for male participants and 27 348 for female participants.

### 3.2 CVD Risk According to HGS

**FIGURE 2:**
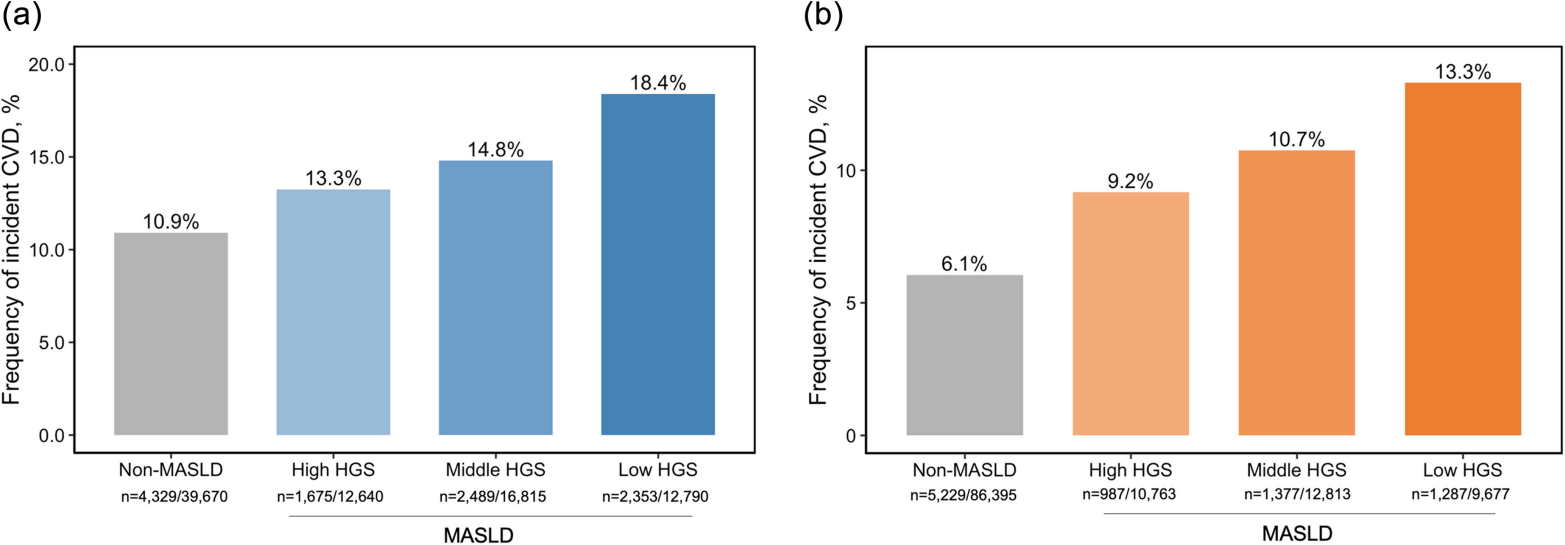
Incidence of cardiovascular events according to handgrip strength in male (a) and female (b) participants. Abbreviations: CVD, cardiovascular disease; HGS, handgrip strength; MASLD, metabolic dysfunction‐associated steatotic liver disease.

Differences in incident CVD events according to HGS are presented in Table 2 and Figure 2. In both male and female cases, the incidence of CVD events increased in the following group order: non‐MASLD, MASLD with high HGS, MASLD with middle HGS, and MASLD with low HGS. The frequency of incident CVD events in male participants was 10.9% for non‐MASLD, 13.3% for MASLD with high HGS, 14.8% for MASLD with middle HGS, and 18.4% for MASLD with low HGS (*p* < 0.001). Similarly, in female cases, the frequency of incident CVD events was 6.1%, 9.2%, 10.7%, and 13.3% for non‐MALSD, MALSD with high HGS, MASLD with middle HGS, and MASLD with low HGS, respectively (*p* < 0.001).

**TABLE 2:** Number and proportion of incident cardiovascular disease events by handgrip strength.

Male (*n* = 81 915)	CVD events	*p*	Female (*n* = 119 648)	CVD events	*p*
Non‐MASLD (*n* = 39 670)	4329 (10.9%)	< 0.001	Non‐MASLD (*n* = 86 395)	5229 (6.1%)	< 0.001
MASLD			MASLD		
High HGS (*n* = 12 640)	1675 (13.3%)		High HGS (*n* = 10 763)	987 (9.2%)	
Middle HGS (*n* = 16 815)	2489 (14.8%)		Middle HGS (*n* = 12 813)	1377 (10.7%)	
Low HGS (*n* = 12 790)	2353 (18.4%)		Low HGS (*n* = 9677)	1287 (13.3%)	

Abbreviations: CVD, cardiovascular disease; HGS, handgrip strength; MASLD, metabolic dysfunction‐associated steatotic liver disease.

**FIGURE 3:**
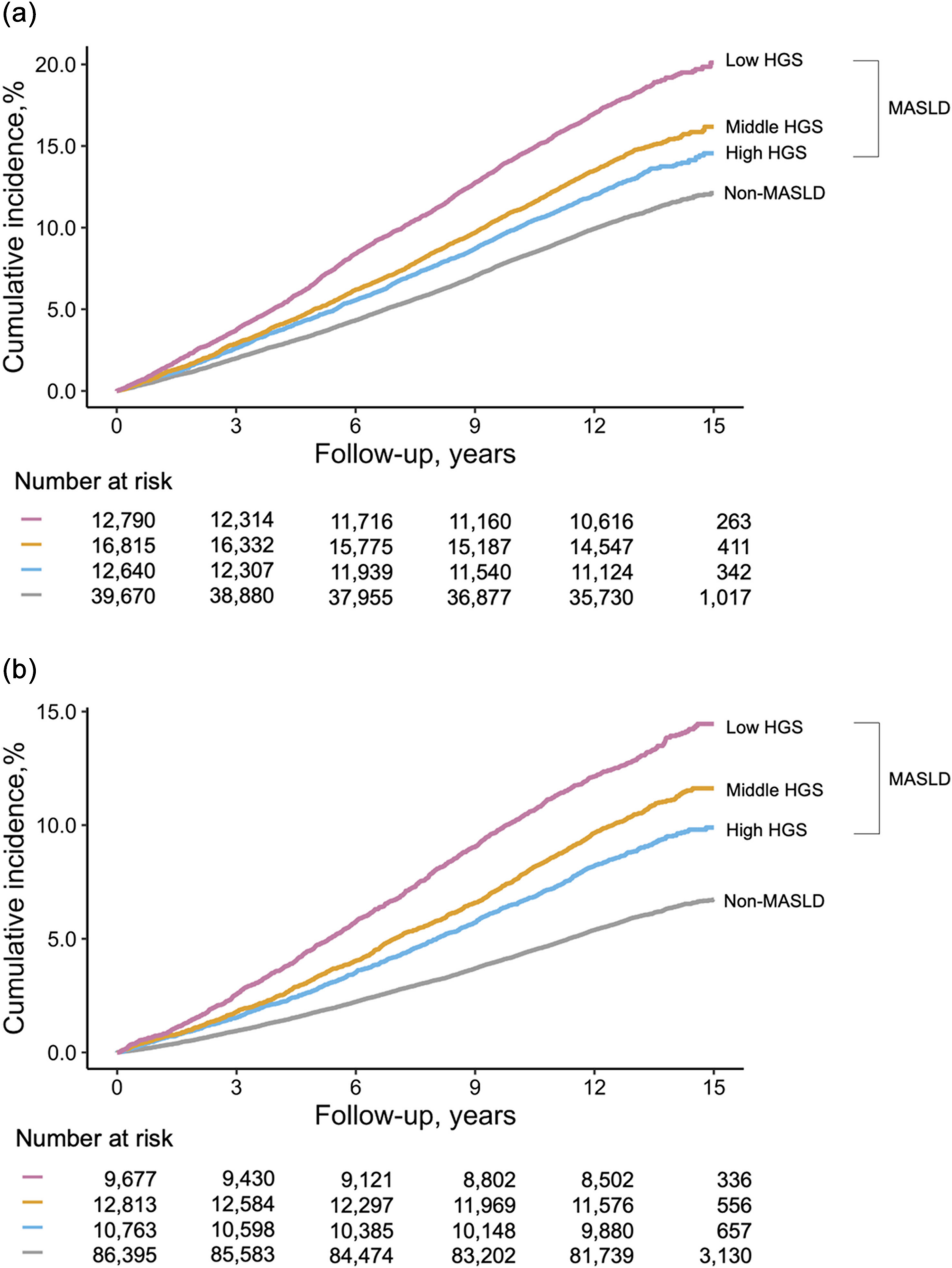
Cumulative incidence of cardiovascular disease events according to handgrip strength in male (a) and female (b) participants. Abbreviations: CVD, cardiovascular disease; HGS, handgrip strength; MASLD, metabolic dysfunction‐associated steatotic liver disease.

In men and women, the cumulative incidence of CVD events increased in the following sequence: non‐MASLD, high, middle, and low HGS in MASLD (Figure 3). In the unadjusted models (model 1, Table 3), the HRs for incident CVD varied according to the HGS in individuals with MASLD. For males, in comparison with the non‐MASLD group, the MASLD group had HRs of 1.23 (95% CI, 1.16–1.30) for high HGS, 1.39 (1.32–1.46) for middle HGS, and 1.77 (1.68–1.86) for low HGS. For females, the corresponding HRs in the MASLD group were 1.52 (1.42–1.63) for high HGS, 1.81 (1.70–1.92) for middle HGS, and 2.28 (2.14–2.42) for low HGS in comparison to the non‐MASLD group. Even after further adjusting for BMI, smoking, diabetes mellitus, hypertension, dyslipidaemia, and physical activity as covariates, statistical significance was maintained, except for MASLD with high HGS for both men and women. The adjusted HRs for incident CVD events in male MASLD participants were 1.03 (0.96–1.10) for high HGS, 1.14 (1.07–1.21) for middle HGS, and 1.38 (1.30–1.46) for low HGS compared with the non‐MALSD group. Among female MASLD participants, the adjusted HRs for incident CVD events were 1.07 (0.97–1.18) for high HGS, 1.25 (1.14–1.37) for middle HGS, and 1.56 (1.43–1.72) for low HGS compared with the non‐MASLD group (model 4, Table 3). We further explored the differences in mortality risk among these groups and found that mortality risks followed trends similar to those observed for CVD events. For males, the mortality rates were as follows: MASLD with high HGS (7.9%), non‐MASLD (8.2%), MASLD with middle HGS (10.4%), and MASLD with low HGS (13.9%). For females, a similar pattern was observed, aligning more closely with the trend seen for CVD incidence. Mortality rates in females increased as follows: non‐MASLD (5.5%), MASLD with high HGS (8.0%), MASLD with middle HGS (8.5%), and MASLD with low HGS (10.7%) (Table S5). The cumulative incidence curve for mortality further illustrates the diverging patterns of mortality based on HGS (Figure S1).

**TABLE 3:** Cardiovascular disease risk according to handgrip strength in male and female participants.

Male participants (*n* = 81 915)	Events	Model 1		Model 2		Model 3		Model 4	
		HR	95% CI	HR	95% CI	HR	95% CI	HR	95% CI
Non‐MASLD (*n* = 39 670)	4329	Reference		Reference		Reference		Reference	
MASLD with high HGS (*n* = 12 640)	1675	1.23	(1.16–1.30)	1.09	(1.03–1.16)	1.02	(0.95–1.08)	1.03	(0.96–1.10)
MASLD with middle HGS (*n* = 16 815)	2489	1.39	(1.32–1.46)	1.21	(1.15–1.27)	1.12	(1.06–1.19)	1.14	(1.07–1.21)
MASLD with low HGS (*n* = 12 790)	2353	1.77	(1.68–1.86)	1.47	(1.39–1.54)	1.36	(1.28–1.45)	1.38	(1.30–1.46)

Female participants (*n* = 119 648)	Events	Model 1		Model 2		Model 3		Model 4	
		HR	95% CI	HR	95% CI	HR	95% CI	HR	95% CI
Non‐MASLD (*n* = 86 395)	5229	Reference		Reference		Reference		Reference	
MASLD with high HGS (*n* = 10 763)	987	1.52	(1.42–1.63)	1.21	(1.13–1.29)	1.11	(1.02–1.21)	1.07	(0.97–1.18)
MASLD with middle HGS (*n* = 12 813)	1377	1.81	(1.70–1.92)	1.42	(1.34–1.51)	1.31	(1.22–1.42)	1.25	(1.14–1.37)
MASLD with low HGS (*n* = 9677)	1287	2.28	(2.14–2.42)	1.72	(1.61–1.83)	1.58	(1.46–1.71)	1.56	(1.43–1.72)

*Note:* Model 1 was unadjusted. Model 2 was adjusted for diabetes mellitus, hypertension, and dyslipidaemia. Model 3 was adjusted for body mass index, smoking, diabetes mellitus, hypertension, and dyslipidaemia. Model 4 was adjusted for body mass index, smoking, diabetes mellitus, hypertension, dyslipidaemia, and physical activity.

Abbreviations: CI, confidence intervals; HR, hazard ratios; HSG, handgrip strength; MASLD, metabolic dysfunction‐associated steatotic liver disease.

### 3.3 CVD Risk According to the Presence of Advanced Liver Fibrosis and HGS

**FIGURE 4:**
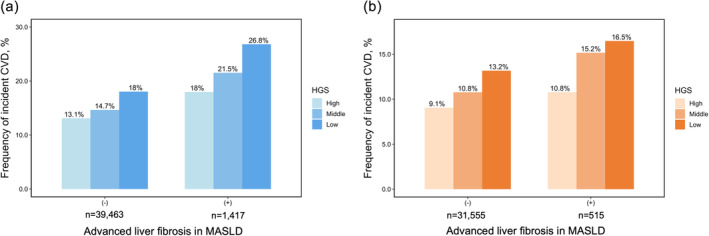
Incidence of cardiovascular disease events according to HGS in MASLD with or without advanced liver fibrosis in male (a) and female (b) participants. Abbreviations: CVD, cardiovascular disease; HGS, handgrip strength; MASLD, metabolic dysfunction‐associated steatotic liver disease.

The incidence of CVD events increased in the following group order: non‐MASLD, MASLD without advanced liver fibrosis, and MASLD with advanced liver fibrosis (Table S6, Figure S2). A tendency for a higher occurrence of CVD events with decreasing HSG was noted regardless of the presence or absence of advanced liver fibrosis in both male and female participants with MASLD (Table 4, Figure 4). The incidence of CVD events according to the presence or absence of advanced liver fibrosis in each HGS group is shown in Figure S3. In the low‐, middle‐ and high‐HGS groups in the MASLD group, the presence of advanced liver fibrosis increased the incidence of CVD events.

**TABLE 4:** Number and proportion of incident CVD events by handgrip strength in MASLD with or without advanced liver fibrosis^a^.

MASLD without advanced liver fibrosis	CVD events	*p*	MASLD with advanced liver fibrosis	CVD events	*p*
Male (*n* = 39 463)			Male (*n* = 1417)		
High HGS (*n* = 11 878)	1556 (13.1%)	< 0.001	High HGS (*n* = 356)	64 (18.0%)	< 0.001
Middle HGS (*n* = 15 706)	2301 (14.7%)		Middle HGS (*n* = 576)	124 (21.5%)	
Low HGS (*n* = 11 879)	2143 (18.0%)		Low HGS (*n* = 485)	130 (26.8%)	
Female (*n* = 31 555)			Female (*n* = 515)		
High HGS (*n* = 10 221)	926 (9.1%)	< 0.001	High HGS (*n* = 167)	18 (10.8%)	< 0.001
Middle HGS (*n* = 12 194)	1313 (10.8%)		Middle HGS (*n* = 178)	27 (15.2%)	
Low HGS (*n* = 9140)	1204 (13.2%)		Low HGS (*n* = 170)	28 (16.5%)	

Abbreviations: CVD, cardiovascular disease; HGS, handgrip strength; MASLD, metabolic dysfunction‐associated steatotic liver disease.

^a^ There are 1365 male and 1183 female MASLD participants for whom the FIB‐4 calculation is impossible due to missing data.

### 3.4 Sensitivity Analysis

The baseline characteristics after the exact PSM process are presented in Table S7. After the exact PSM, the incidence of CVD remained consistent in the following order: non‐MASLD, MASLD with high HGS, MASLD with middle HGS, and MASLD with low HGS (Table S8). In both unadjusted and fully adjusted models of the matched cohorts, the risk of CVD events showed an increasing trend with lower HGS in both males and females (Table S9).

To determine whether low HGS increases CVD risk in the non‐MASLD group, a stratified analysis was performed. This analysis examined the incidence of CVD events based on HGS levels within the non‐MASLD group. The results indicated that, as in the MASLD group, lower HGS was associated with a higher risk of CVD. For males in the non‐MASLD group, the incidence of CVD increased as follows: high HGS (9.2%), middle HGS (10.6%), and low HGS (12.9%). A similar pattern was observed among females, with CVD incidence increasing from high HGS (5.3%) to middle HGS (6.1%) and low HGS (6.8%) (Table S10). In the fully adjusted model, these associations remained consistent, with high HGS as the reference group. For male participants, the HRs were 1.35 (1.24–1.45) for low HGS and 1.14 (1.05–1.23) for middle HGS. Similarly, for female participants, the HRs were 1.25 (1.17–1.34) for low HGS and 1.15 (1.08–1.23) for middle HGS (Table S11). These findings demonstrate that low HGS is a significant predictor of CVD risk even in the absence of MASLD. An additional sensitivity analysis was conducted to further assess the association between HGS and CVD risk across both MASLD and non‐MASLD populations. The results, presented in Table S12, showed a consistent trend in which lower HGS was associated with a higher risk of CVD in both groups. Among males, the risk of CVD increased as HGS decreased, and this trend was more evident in individuals with MASLD. A similar pattern was observed among females, where lower HGS was linked to a progressively higher CVD risk. The highest CVD risk was observed in individuals with both MASLD and low HGS, indicating that the combination of these factors contributes to a greater risk than either condition alone.

## 4 Discussion

In this large‐scale, longitudinal follow‐up study, we further confirmed that the incident CVD risk might differ according to HGS in participants with MASLD, demonstrating that CVD risk increased in the following order: non‐MASLD, MASLD with high HGS, MASLD with middle HGS, and MASLD with low HGS. Even in the model fully adjusted for confounders, individuals without MASLD and those with MASLD and high HGS exhibited similar and relatively low CVD risks. This was followed by MASLD with middle HGS and then those with low HGS, in ascending order of incident CVD risk.

A large prospective study demonstrated that low muscle strength increases CVD risk in the general population [10], and other studies corroborated this result [11, 12]. HGS is a representative method that allows the measurement of muscle strength in a cost‐effective, easy, and rapid manner [16]. In the present study, using HGS values, we revealed that in participants with MASLD, the CVD risk increases as HGS decreases. Recently, the importance of sarcopenia in predicting MASLD prognosis has been emphasized, and several studies have shown that low muscle mass in patients with NAFLD increases the atherosclerotic cardiovascular disease (ASCVD) risk score [17, 21]. A recent study has also revealed that low HGS increases the ASCVD risk score in metabolic dysfunction‐associated fatty liver disease [25]. However, these studies were cross‐sectional in design, and notably, there is almost no longitudinal data on how incident CVD events change according to HGS in MASLD, where HGS is a reflection of muscle strength.

The connection between HGS and CVD can be attributed to the role of HGS as a marker of systemic health, where diminished muscle strength is linked to higher levels of inflammatory markers and insulin resistance, which are critical factors in CVD development [26]. Furthermore, as a measure of sarcopenia and metabolic health, HGS might reflect vascular function and arterial stiffness, factors that remarkably increase CVD risk [10]. We noted a clear gradient in the incidence of CVD events, with the lowest risk noted in the non‐MASLD, followed by the MASLD with high HGS, MASLD with middle HGS, and the highest risk in the MASLD with low HGS. This indicates that while MASLD increases the risk of CVD, the risk of CVD varies according to HGS. Furthermore, CVD risk was comparable between individuals without MASLD and those with MASLD who had a high HGS across fully adjusted models, irrespective of sex. This suggests that enhancing muscle strength may serve as a preventive strategy for individuals with MASLD. However, this hypothesis warrants further investigation through interventional studies.

Owing to diet changes and the increase in sedentary lifestyle habits, the prevalence of MASLD is rising [27, 28]. The acceleration of aging society and escalation of such chronic metabolic diseases can lead to an increase in CVD incidence [29], rendering CVD in patients with MASLD a significant public health threat [5]. Thus, identifying the high‐risk group for CVD within MASLD is critical, not only at individual levels but society levels. The ASCVD risk score, which is widely employed to predict CVD risk, has been developed for the general population [17, 21]. The ASCVD risk score includes age, diabetes mellitus, sex, smoking, cholesterol, HDL cholesterol, systolic blood pressure, hypertension treatment, and race, and has been criticized for possibly overestimating CVD events [30]. In our study, HGS appeared to be an independent risk factor for CVD in MASLD, indicating that if a CVD risk prediction model is developed specifically for MASLD in the future, HGS, which can reflect muscle strength, is expected to play a crucial role.

Our study further elucidates the intricate relationship between HGS, advanced liver fibrosis, and CVD risk in MASLD. This reinforces the notion that a lower HGS is a significant predictor of CVD risk, irrespective of advanced liver fibrosis. The presence of advanced liver fibrosis amplifies this risk, even among individuals with comparable HGS levels, which is consistent with recent findings that verify the role of liver fibrosis in elevating CVD risk [31, 32]. This indicates a multifaceted impact on cardiovascular health, where both HGS and liver fibrosis might independently and synergistically contribute to CVD risk via mechanisms such as endothelial dysfunction, exacerbated by factors such as vascular impairment and inflammatory responses [33].

One of the strengths of this study is its longitudinal nature via a large prospective cohort to examine the relationship between muscle strength and CVD in patients with MASLD with validated tools for measuring muscle strength; however, but it also has several limitations. First, hepatic steatosis was defined using the FLI, a non‐invasive score, instead of histology or imaging. However, this method is thoroughly validated [34], and international guidelines indicate non‐invasive indicators, such as the FLI, are considered acceptable substitutes for identifying hepatic steatosis in epidemiological research [20, 35]. Furthermore, in recent studies utilizing large cohorts, hepatic steatosis has also been defined using the FLI [4, 5, 19]. Second, owing to the absence of liver biopsy data, FIB‐4 was used to define advanced liver fibrosis. Liver biopsy is the gold standard for the diagnosis of liver fibrosis. However, liver biopsy is an invasive procedure and can have complications; thus, it is not commonly performed solely for the diagnosis of liver fibrosis [36]. Although it is known to be less accurate than non‐invasive methods such as transient elastography or magnetic resonance elastography, FIB‐4 is a non‐invasive scoring system recommended by most guidelines as the first step to differentiate advanced liver fibrosis [37]. Furthermore, in practice, numerous studies have used FIB‐4 to diagnose advanced liver fibrosis in NAFLD [17, 31, 32]. Third, while HGS serves as a simple and valuable measure of muscle strength, it does not encompass all dimensions of musculoskeletal health. It primarily evaluates the strength of the upper extremities and may not reflect an individual's overall muscular condition or functional capabilities. Fourth, although the analyses were stratified by age and sex, which significantly affect HGS, and adjusted for various covariates, other factors that may influence HGS, such as nutritional status, psychological factors, and environmental conditions, were not assessed. This omission may have introduced bias into our analyses [38, 39, 40].

In conclusion, this large prospective cohort study utilizing UK Biobank data demonstrated that in MASLD, low muscle strength was associated with an increased risk of CVD, regardless of disease severity. This observation indicates the importance of investigating whether interventions for enhancing muscle strength could effectively decrease CVD risk in patients with MASLD. However, further prospective studies are required to explore this potential.

## Conflicts of Interest

The authors declare no conflicts of interest.

## Supporting information


**Table S1** Diagnoses corresponding to other liver diseases or alcohol or drug use disorders.
**Table S2.** Definition of past disease at baseline.
**Table S3.** Definitions of hepatic steatosis and advanced liver fibrosis.
**Table S4.** Baseline characteristics according to HGS in participants with MASLD.
**Table S5.** Mortality outcome according to handgrip strength.
**Table S6.** Number and proportion of incident CVD events in non‐MASLD and MASLD with or without advanced liver fibrosis*.
**Table S7.** Baseline characteristics of the study population after exact propensity score matching.
**Table S8.** Number and proportion of incident cardiovascular disease events by handgrip strength after exact propensity score matching.
**Table S9.** Cardiovascular disease risk according to handgrip strength in male and female participants after propensity score matching.
**Table S10.** Incident cardiovascular disease events according to handgrip strength among non‐MASLD group.
**Table S11.** Cardiovascular disease risk according to handgrip strength in male and female participants in non‐MASLD group.
**Table S12.** Cardiovascular disease risk according to handgrip strength in male and female participants in non‐MASLD and MASLD group.
**Figure S1.** Cumulative incidence of mortality events according to handgrip strength in male (a) and female (b) subjects.
**Figure S2.** Incidence of CVD events in non‐MASLD participants and in MASLD participants with or without advanced liver fibrosis, for males (a) and females (b). CVD, cardiovascular disease; MASLD, metabolic dysfunction‐associated steatotic liver disease.
**Figure S3.** Incidence of CVD events by the presence or absence of advanced liver fibrosis within each HGS group for males (A) and females (B) in MASLD. CVD, cardiovascular disease; MASLD, metabolic dysfunction‐associated steatotic liver disease; HGS, handgrip strength.
