# Supplementary material for: Association Between Handgrip Strength and Cardiovascular Disease Risk in MASLD: A Prospective Study From UK Biobank
Source: J Cachexia Sarcopenia Muscle. 2025 Mar 4;16(2):e13757. doi: 10.1002/jcsm.13757 (PMC11876860; doi:10.1002/jcsm.13757)
Supplement: Supplementary file 1 — Table S1 Diagnoses corresponding to other liver diseases or alcohol or drug use disorders. Table S2. Definition of past disease at baseline. Table S3. Definitions of hepatic steatosis and advanced liver fibrosis. Table S4. Baseline characteristics according to HGS in participants with MASLD. Table S5. Mortality outcome according to handgrip strength. Table S6. Number and proportion of incident CVD events in non‐MASLD and MASLD with or without advanced liver fibrosis*. Table S7. Baseline characteristics of the study population after exact propensity score matching. Table S8. Number and proportion of incident cardiovascular disease events by handgrip strength after exact propensity score matching. Table S9. Cardiovascular disease risk according to handgrip strength in male and female participants after propensity score matching. Table S10. Incident cardiovascular disease events according to handgrip strength among non‐MASLD group. Table S11. Cardiovascular disease risk according to handgrip strength in male and female participants in non‐MASLD group. Table S12. Cardiovascular disease risk according to handgrip strength in male and female participants in non‐MASLD and MASLD group. Figure S1. Cumulative incidence of mortality events according to handgrip strength in male (a) and female (b) subjects. Figure S2. Incidence of CVD events in non‐MASLD participants and in MASLD participants with or without advanced liver fibrosis, for males (a) and females (b). CVD, cardiovascular disease; MASLD, metabolic dysfunction‐associated steatotic liver disease. Figure S3. Incidence of CVD events by the presence or absence of advanced liver fibrosis within each HGS group for males (A) and females (B) in MASLD. CVD, cardiovascular disease; MASLD, metabolic dysfunction‐associated steatotic liver disease; HGS, handgrip strength. [file JCSM-16-e13757-s001.docx]

**Supplementary materials**

**Table S1.** Diagnoses corresponding to other liver diseases or alcohol or drug use disorders

| Diagnosis | ICD-10 |
| --- | --- |
| Other liver diseases  Alcoholic liver disease | K70 |
| Viral Hepatitis  Autoimmune liver disease  Chronic hepatitis, unspecified  Wilson disease  Hemochromatosis  Budd-Chari syndrome  Alpha-1-antitrypsin deficiency  Secondary or unspecified biliary cirrhosis | B16, B17, B18, B19  K830, K743, K745, K754  K739, K732  K830  E831  I820, K765  E880  K744, K745 |
| Alcohol or drug use disorder  Alcohol use disorder  Drug use disorder | F10, E244, G621, I426, K292, G312, G721, K852, K860, T510, T519, X65, Y573, Z502, Z714, Z721  F11, F12, F13, F14, F16, F18, F19 |

**Table S2.** Definition of past disease at baseline

| Diagnosis | Definition |
| --- | --- |
| Hypertension |  |
| Self-reported  ICD-10  Dyslipidemia  Self-reported  Diabetes mellitus  Self-reported  ICD-10 | Field 6153 (Medication for cholesterol, blood pressure, diabetes, or take exogenous hormones)  Field 6177 (Medication for cholesterol, blood pressure or diabetes)  I10 (Essential (primary) hypertension)  Field 6153 (Medication for cholesterol, blood pressure, diabetes, or take exogenous hormones)  Field 6177 (Medication for cholesterol, blood pressure or diabetes)  Field 6153 (Medication for cholesterol, blood pressure, diabetes, or take exogenous hormones)  Field 6177 (Medication for cholesterol, blood pressure or diabetes)  E11 (non-insulin-dependent diabetes mellitus)  E12 (malnutrition-related diabetes mellitus)  E13 (other specified diabetes mellitus)  E14 (unspecified diabetes mellitus) |

**Table S3.** Definitions of hepatic steatosis and advanced liver fibrosis

| Definition | Criteria |
| --- | --- |
| Hepatic steatosis | Fatty liver index (FLI) ≥60  FLI = 1 / (1 + e^–^*^x^*) × 100; where  *x* = 0.953 × log_e_ *triglyceride* + 0.139 × *body mass index*  + 0.718 × log_e_ *γ-glutamyl-transferase* + 0.053 × *waist circumference*  − 15.745 |
| Advanced liver fibrosis | FIB-4 >2.67  FIB-4 = (Age x AST)/(Platelet count x √ALT) |

AST, aspartate aminotransferase; ALT, alanine aminotransferase

| **Table S4.** Baseline characteristics according to HGS in participants with MASLD | | | | | | | | |
| --- | --- | --- | --- | --- | --- | --- | --- | --- |
| Variables | Male | | | | Female | | | |
|  | Low HGS | Middle HGS | High HGS | *P* | Low HGS | Middle HGS | High HGS | *P* |
| n | 24,937 | 32,728 | 24,250 |  | 33,088 | 47,194 | 39,366 |  |
| Age, years | 57.1 ± 8.4 | 56.4 ± 8.3 | 56.0 ± 8.2 | <0.001 | 56.3 ± 8.3 | 56.8 ± 8.2 | 56.5 ± 7.7 | <0.001 |
| Age (%) |  |  |  | <0.001 |  |  |  | <0.001 |
| under 50 | 5,888 (23.6) | 8,279 (25.3) | 6,127 (25.3) |  | 8,784 (26.5) | 11,184 (23.7) | 7,715 (19.6) |  |
| 50–60 | 7,502 (30.1) | 10,236 (31.3) | 7,598 (31.3) |  | 10,808 (32.7) | 14,487 (30.7) | 14,476 (36.8) |  |
| 60–70 | 11,401 (45.7) | 14,066 (43.0) | 10,379 (42.8) |  | 13,331 (40.3) | 21,283 (45.1) | 17,030 (43.3) |  |
| over 70 | 146 (0.6) | 147 (0.4) | 146 (0.6) |  | 165 (0.5) | 240 (0.5) | 145 (0.4) |  |
| Race (%) |  |  |  | <0.001 |  |  |  | <0.001 |
| White | 21,147 (84.8) | 29,988 (91.6) | 22,685 (93.5) |  | 28,766 (86.9) | 43,450 (92.1) | 36,639 (93.1) |  |
| Black | 719 (2.9) | 768 (2.3) | 702 (2.9) |  | 798 (2.4) | 1,150 (2.4) | 1,283 (3.3) |  |
| Asian | 2,221 (8.9) | 1,192 (3.6) | 385 (1.6) |  | 2,365 (7.1) | 1,417 (3.0) | 539 (1.4) |  |
| Others | 850 (3.4) | 780 (2.4) | 478 (2.0) |  | 1,159 (3.5) | 1,177 (2.5) | 905 (2.3) |  |
| Smoking (%) |  |  |  | <0.001 |  |  |  | <0.001 |
| Never | 13,007 (52.5) | 16,896 (51.8) | 12,424 (51.4) |  | 20,993 (63.8) | 29,284 (62.3) | 23,887 (60.9) |  |
| Previous | 8,513 (34.3) | 11,714 (35.9) | 8,944 (37.0) |  | 8,842 (26.9) | 13,645 (29.0) | 11,996 (30.6) |  |
| Current | 3,275 (13.2) | 4,007 (12.3) | 2,799 (11.6) |  | 3,079 (9.4) | 4,100 (8.7) | 3,368 (8.6) |  |
| Diabetes mellitus (%) | 2,523 (10.1) | 2,173 (6.6) | 1,224 (5.0) | <0.001 | 1,872 (5.7) | 1,847 (3.9) | 1,350 (3.4) | <0.001 |
| Hypertension (%) | 8,104 (32.5) | 9,424 (28.8) | 6,573 (27.1) | <0.001 | 8,449 (25.5) | 11,167 (23.7) | 9,249 (23.5) | <0.001 |
| Dyslipidemia (%) | 5,514 (22.1) | 6,068 (18.5) | 4,086 (16.8) | <0.001 | 4,721 (14.3) | 5,765 (12.2) | 4,420 (11.2) | <0.001 |
| Body mass index, kg/m^2^ | 27.6 ± 4.6 | 27.7 ± 4.2 | 28.0 ± 4.1 | <0.001 | 27.5 ± 5.6 | 27.2 ± 5.3 | 27.5 ± 5.4 | <0.001 |
| Waist circumference, cm | 96.8 ± 12.2 | 96.4 ± 11.4 | 96.9 ± 11.0 | <0.001 | 85.8 ± 13.4 | 85.0 ± 12.8 | 85.6 ± 12.9 | <0.001 |
| Physical activity (%)* |  |  |  | <0.001 |  |  |  | <0.001 |
| <600 | 4,407 (17.7) | 4,897 (15.0) | 3,425 (14.1) |  | 5,515 (16.7) | 6,399 (13.6) | 4,888 (12.4) |  |
| 600-3000 | 10,018 (40.2) | 13,342 (40.8) | 9,839 (40.6) |  | 12,458 (37.7) | 18,633 (39.5) | 1,5734 (40.0) |  |
| ≥3000 | 5,973 (24.0) | 9,431 (28.8) | 7,591 (31.3) |  | 6,780 (20.5) | 11,345 (24.0) | 10,548 (26.8) |  |
| Systolic blood pressure, mmHg | 141.5 ± 18.9 | 142.8 ± 18.5 | 143.9 ± 18.1 | <0.001 | 136.1 ± 20.5 | 137.9 ± 20.5 | 139.4 ± 20.3 | <0.001 |
| Diastolic blood pressure, mmHg | 83.2 ± 10.6 | 84.1 ± 10.4 | 84.9 ± 10.4 | <0.001 | 80.0 ± 10.6 | 80.7 ± 10.5 | 81.6 ± 10.5 | <0.001 |
| Handgrip strength, kg | 30.8 ± 5.1 | 41.0 ± 3.2 | 50.8 ± 5.3 | <0.001 | 17.2 ± 4.0 | 24.1 ± 2.9 | 30.9 ± 3.8 | <0.001 |
| Platelet count, 10^9^/L | 234.2 ± 57.2 | 231.5 ± 54.3 | 229.7 ± 52.3 | <0.001 | 264.4 ± 62.4 | 261.0 ± 59.6 | 259.1 ± 58.3 | <0.001 |
| Aspartate aminotransferase, U/L | 27.9 ± 11.6 | 27.9 ± 10.9 | 28.1 ± 9.8 | 0.011 | 24.6 ± 9.6 | 24.6 ± 10.0 | 24.6 ± 8.5 | 0.85 |
| Alanine aminotransferase, U/L | 26.9 ± 15.7 | 26.7 ± 14.1 | 27.2 ± 15.0 | 0.001 | 20.2 ± 12.2 | 20.1 ± 12.4 | 20.1 ± 11.2 | 0.342 |
| Albumin, g/dL | 4.5 ± 0.3 | 4.6 ± 0.3 | 4.6 ± 0.3 | <0.001 | 4.5 ± 0.3 | 4.5 ± 0.3 | 4.5 ± 0.3 | <0.001 |
| Total bilirubin, mg/dL | 0.6 ± 0.3 | 0.6 ± 0.3 | 0.6 ± 0.3 | <0.001 | 0.5 ± 0.2 | 0.5 ± 0.2 | 0.5 ± 0.2 | <0.001 |
| Gamma glutamyl transferase, U/L | 44.7 ± 49.7 | 42.9 ± 43.2 | 41.0 ± 37.7 | <0.001 | 31.0 ± 34.6 | 29.5 ± 31.5 | 28.7 ± 29.0 | <0.001 |
| Creatinine, mg/dL | 0.81 ± 0.25 | 0.82 ± 0.19 | 0.83 ± 0.14 | <0.001 | 0.63 ± 0.18 | 0.64 ± 0.13 | 0.66 ± 0.12 | <0.001 |
| Glucose, mg/dL | 95.5 ± 28.2 | 93.9 ± 24.1 | 92.8 ± 20.0 | <0.001 | 92.7 ± 22.0 | 92.2 ± 19.2 | 91.9 ± 17.7 | <0.001 |
| Hemoglobin A1c, % | 5.6 ± 0.8 | 5.5 ± 0.7 | 5.4 ± 0.6 | <0.001 | 5.5 ± 0.6 | 5.5 ± 0.6 | 5.4 ± 0.5 | <0.001 |
| Total cholesterol, mg/dL | 209.4 ± 43.7 | 213.8 ± 42.6 | 215.0 ± 41.8 | <0.001 | 224.9 ± 43.9 | 227.9 ± 43.7 | 228.5 ± 43.6 | <0.001 |
| HDL-cholesterol, mg/dL | 48.8 ± 12.2 | 49.5 ± 12.1 | 49.3 ± 11.6 | <0.001 | 59.6 ± 14.4 | 60.8 ± 14.5 | 60.9 ± 14.5 | <0.001 |
| LDL-cholesterol, mg/dL | 133.0 ± 33.4 | 136.3 ± 32.6 | 137.5 ± 31.9 | <0.001 | 139.5 ± 33.8 | 141.3 ± 33.7 | 141.9 ± 33.6 | <0.001 |
| Triglycerides, mg/dL | 174.5 ± 102.3 | 173.7 ± 100.8 | 172.1 ± 100.1 | 0.026 | 144.1 ± 80.6 | 140.5 ± 77.5 | 138.3 ± 75.5 | <0.001 |
| Values are showed as frequency (%) or mean ± standard deviation.  HGS, handgrip strength; MASLD, metabolic dysfunction-associated steatotic liver disease; HDL, high-density lipoprotein; LDL, low-density lipoprotein.  * Physical activity is presented as metabolic equivalent of task minutes per week. There are 12,992 missing data for male participants, and 27,348 for female participants. | | | | | | | | |

**Table S5.** Mortality outcome according to handgrip strength

| Male (n=81,915) | Mortality | *P* | Female (n=119,648) | Mortality | *P* |
| --- | --- | --- | --- | --- | --- |
| Non-MASLD (n=39,670) | 3,238 (8.2%) | <0.001 | Non-MASLD (n=86,395) | 4,718 (5.5%) | <0.001 |
| MASLD |  |  | MASLD |  |  |
| High HGS (n=12,640) | 1,002 (7.9%) |  | High HGS (n=10,763) | 864 (8.0%) |  |
| Middle HGS (n=16,815) | 1,746 (10.4%) |  | Middle HGS (n=12,813) | 1,084 (8.5%) |  |
| Low HGS (n=12,790) | 1,782 (13.9%) |  | Low HGS (n=9,677) | 1,034 (10.7%) |  |
| MALSD, metabolic dysfunctiin-associated steatotic liver disease; HGS, handgrip strength | | | | | |

| **Table S6.** Number and proportion of incident CVD events in non-MASLD and MASLD with or without advanced liver fibrosis* | | |  |
| --- | --- | --- | --- |
| Male (n=79,389) | CVD events | *P* |  |
| Non-MASLD (n= 38,509) | 4,194 (10.9%) | <0.001 |  |
| MASLD without advanced liver fibrosis (n=39,463) | 6,000 (15.2%) |  |  |
| MASLD with advanced liver fibrosis (n= 1,417) | 318 (22.4%) |  |  |
| Female (n=115,566) |  |  |  |
| Non-MASLD (n= 83,496) | 5,035 (6.0%) | <0.001 |  |
| MASLD without advanced liver fibrosis (n=31,555) | 3,443 (10.9%) |  |  |
| MASLD with advanced liver fibrosis (n=515) | 73 (14.2%) |  |  |
| CVD, cardiovascular disease; MASLD, Metabolic dysfunction-associated steatotic liver disease  *There are 2,526 male and 4,082 female NAFLD patients for whom the FIB-4 calculation is impossible due to missing data. | | |  |

**Table S7.** Baseline characteristics of the study population after exact propensity score matching

| Variables | Male | | |  |  | Female | | |  |  |
| --- | --- | --- | --- | --- | --- | --- | --- | --- | --- | --- |
|  | Overall | Non-MASLD | MASLD | *P* | *SMD* | Overall | Non-MASLD | MASLD | *P* | *SMD* |
| n | 53,588 | 26,794 | 26,794 |  |  | 48,832 | 24,416 | 24,416 |  |  |
| Age, years | 56.3 ± 8.4 | 56.4 ± 8.4 | 56.3 ± 8.3 | 0.223 | 0.011 | 57.7 ± 7.7 | 57.7 ± 7.7 | 57.7 ± 7.6 | 0.376 | 0.008 |
| Age (%) |  |  |  | 0.99 | <0.001 |  |  |  | 0.99 | <0.001 |
| under 50 | 13,584 (25.3) | 6,792 (25.3) | 6,792 (25.3) |  |  | 8,574 (17.6) | 4,287 (17.6) | 4,287 (17.6) |  |  |
| 50–60 | 16,618 (31.0) | 8,309 (31.0) | 8,309 (31.0) |  |  | 16,396 (33.6) | 8,198 (33.6) | 8,198 (33.6) |  |  |
| 60–70 | 23,138 (43.2) | 11,569 (43.2) | 11,569 (43.2) |  |  | 23,646 (48.4) | 11,823 (48.4) | 11,823 (48.4) |  |  |
| Over 70 | 248 (0.5) | 124 (0.5) | 124 (0.5) |  |  | 216 (0.4) | 108 (0.4) | 108 (0.4) |  |  |
| Race |  |  |  | 0.99 | <0.001 |  |  |  | 0.99 | <0.001 |
| White | 48,542 (90.6) | 24,271 (90.6) | 24,271 (90.6) |  |  | 44,510 (91.1) | 22,255 (91.1) | 22,255 (91.1) |  |  |
| Black | 1,144 (2.1) | 572 (2.1) | 572 (2.1) |  |  | 1,478 (3.0) | 739 (3.0) | 739 (3.0) |  |  |
| Asian | 2,496 (4.7) | 1,248 (4.7) | 1,248 (4.7) |  |  | 1,614 (3.3) | 807 (3.3) | 807 (3.3) |  |  |
| Others | 1,406 (2.6) | 703 (2.6) | 703 (2.6) |  |  | 1,230 (2.5) | 615 (2.5) | 615 (2.5) |  |  |
| Smoking (%) |  |  |  | 0.99 | <0.001 |  |  |  | 0.99 | <0.001 |
| Never | 27,152 (50.7) | 13,576 (50.7) | 13,576 (50.7) |  |  | 29,116 (59.6) | 14,558 (59.6) | 14,558 (59.6) |  |  |
| Previous | 19,612 (36.6) | 9,806 (36.6) | 9,806 (36.6) |  |  | 15,278 (31.3) | 7,639 (31.3) | 7,639 (31.3) |  |  |
| Current | 6,824 (12.7) | 3,412 (12.7) | 3,412 (12.7) |  |  | 4,438 (9.1) | 2,219 (9.1) | 2,219 (9.1) |  |  |
| Diabetes mellitus (%) | 2,444 (4.6) | 1,222 (4.6) | 1,222 (4.6) | 0.99 | <0.001 | 2,386 (4.9) | 1,193 (4.9) | 1,193 (4.9) | 0.99 | <0.001 |
| Hypertension (%) | 12,942 (24.2) | 6,471 (24.2) | 6,471 (24.2) | 0.99 | <0.001 | 18,368 (37.6) | 9,184 (37.6) | 9,184 (37.6) | 0.99 | <0.001 |
| Dyslipidemia (%) | 8,278 (15.4) | 4,139 (15.4) | 4,139 (15.4) | 0.99 | <0.001 | 8,734 (17.9) | 4,367 (17.9) | 4,367 (17.9) | 0.99 | <0.001 |
| Body mass index, kg/m^2^ | 27.5 ± 4.1 | 24.9 ± 2.4 | 30.2 ± 3.7 | <0.001 | 1.694 | 29.4 ± 5.8 | 25.3 ± 3.2 | 33.5 ± 4.8 | <0.001 | 2.005 |
| Waist circumference, cm | 96.0 ± 10.9 | 88.9 ± 6.9 | 103.2 ± 9.3 | <0.001 | 1.743 | 90.4 ± 13.5 | 80.5 ± 8.3 | 100.3 ± 10.0 | <0.001 | 2.151 |
| Physical activity (%)* | |  |  | <0.001 | 0.165 |  |  |  | <0.001 | 0.053 |
| <600 | 7,150 (13.3) | 3,785 (14.1) | 3,365 (12.6) |  |  | 8,470 (17.3) | 4,224 (17.3) | 4,246 (17.4) |  |  |
| 600–3000 | 20,745 (38.7) | 11,057 (41.3) | 9,688 (36.2) |  |  | 17,739 (36.3) | 9,087 (37.2) | 8,652 (35.4) |  |  |
| ≥3000 | 15,720 (29.3) | 7,742 (28.9) | 7,978 (29.8) |  |  | 9,064 (18.6) | 4,597 (18.8) | 4,467 (18.3) |  |  |
| Systolic blood pressure, mmHg | 142.5 ± 18.5 | 140.2 ± 18.8 | 144.7 ± 18.0 | <0.001 | 0.246 | 141.7 ± 20.4 | 139.8 ± 21.2 | 143.6 ± 19.4 | <0.001 | 0.183 |
| Diastolic blood pressure, mmHg | 84.0 ± 10.4 | 81.8 ± 10.3 | 86.1 ± 10.0 | <0.001 | 0.422 | 82.9 ± 10.6 | 80.6 ± 10.6 | 85.2 ± 10.1 | <0.001 | 0.438 |
| Handgrip strength, kg | 40.9 ± 9.0 | 40.5 ± 8.7 | 41.2 ± 9.2 | <0.001 | 0.081 | 24.0 ± 6.4 | 24.1 ± 6.2 | 23.8 ± 6.6 | <0.001 | 0.039 |
| Handgrip strength (%) | |  |  | <0.001 | 0.042 |  |  |  | 0.038 | 0.023 |
| High | 15,928 (29.7) | 7,726 (28.8) | 8,202 (30.6) |  |  | 15,832 (32.4) | 7,903 (32.4) | 7,929 (32.5) |  |  |
| Middle | 21,566 (40.2) | 10,828 (40.4) | 10,738 (40.1) |  |  | 19,223 (39.4) | 9,733 (39.9) | 9,490 (38.9) |  |  |
| Low | 16,094 (30.0) | 8,240 (30.8) | 7,854 (29.3) |  |  | 13,777 (28.2) | 6,780 (27.8) | 6,997 (28.7) |  |  |
| Platelet count, 10^9^/L | 232.0 ± 54.5 | 231.2 ± 55.4 | 232.8 ± 53.5 | 0.001 | 0.029 | 264.9 ± 60.9 | 257.6 ± 58.9 | 272.2 ± 61.9 | <0.001 | 0.242 |
| Aspartate aminotransferase, U/L | 27.8 ± 10.1 | 26.2 ± 8.8 | 29.4 ± 11.1 | <0.001 | 0.327 | 25.3 ± 9.7 | 24.0 ± 7.0 | 26.5 ± 11.7 | <0.001 | 0.264 |
| Alanine aminotransferase, U/L | 26.7 ± 14.5 | 21.8 ± 9.7 | 31.5 ± 16.8 | <0.001 | 0.699 | 22.0 ± 13.5 | 18.4 ± 9.1 | 25.5 ± 16.0 | <0.001 | 0.549 |
| Albumin, g/dL | 4.6 ± 0.3 | 4.6 ± 0.3 | 4.6 ± 0.3 | 0.025 | 0.019 | 4.5 ± 0.3 | 4.5 ± 0.3 | 4.4 ± 0.3 | <0.001 | 0.288 |
| Total bilirubin, mg/dL | 0.6 ± 0.3 | 0.6 ± 0.3 | 0.6 ± 0.3 | <0.001 | 0.136 | 0.5 ± 0.2 | 0.5 ± 0.2 | 0.4 ± 0.2 | <0.001 | 0.235 |
| Gamma glutamyl transferase, U/L | 42.0 ± 41.7 | 29.4 ± 19.7 | 54.6 ± 52.6 | <0.001 | 0.635 | 35.0 ± 37.9 | 24.3 ± 17.6 | 45.7 ± 48.3 | <0.001 | 0.589 |
| Creatinine, mg/dL | 0.8 ± 0.2 | 0.8 ± 0.2 | 0.8 ± 0.2 | <0.001 | 0.066 | 0.7 ± 0.2 | 0.6 ± 0.2 | 0.7 ± 0.1 | <0.001 | 0.052 |
| Glucose, mg/dL | 92.8 ± 21.2 | 91.7 ± 19.3 | 93.9 ± 22.9 | <0.001 | 0.105 | 93.5 ± 20.8 | 92.1 ± 19.3 | 94.9 ± 22.1 | <0.001 | 0.135 |
| Hemoglobin A1c, % | 5.4 ± 0.6 | 5.4 ± 0.6 | 5.5 ± 0.7 | <0.001 | 0.212 | 5.5 ± 0.6 | 5.4 ± 0.5 | 5.6 ± 0.7 | <0.001 | 0.292 |
| Total cholesterol, mg/dL | 215.6 ± 42.2 | 208.8 ± 39.9 | 222.5 ± 43.4 | <0.001 | 0.329 | 228.8 ± 45.0 | 224.1 ± 42.9 | 233.6 ± 46.5 | <0.001 | 0.213 |
| HDL-cholesterol, mg/dL | 49.5 ± 11.9 | 53.2 ± 12.4 | 45.9 ± 10.2 | <0.001 | 0.646 | 58.0 ± 14.0 | 63.1 ± 14.4 | 53.0 ± 11.5 | <0.001 | 0.78 |
| LDL-cholesterol, mg/dL | 137.8 ± 32.4 | 132.2 ± 31.1 | 143.4 ± 32.8 | <0.001 | 0.35 | 143.1 ± 34.7 | 137.5 ± 33.2 | 148.8 ± 35.3 | <0.001 | 0.33 |
| Triglycerides, mg/dL | 172.3 ± 98.9 | 124.6 ± 56.1 | 220.1 ± 108.8 | <0.001 | 1.103 | 159.5 ± 86.0 | 122.2 ± 56.2 | 196.9 ± 94.1 | <0.001 | 0.965 |
| Values are shown as frequency (%) or mean ± standard deviation. | | | |  |  |  |  |  |  |  |
| MASLD, metabolic dysfunction-associated steatotic liver disease; SMD, standardized mean difference; HDL, high-density lipoprotein; LDL, low-density lipoprotein. | | | | | | | | |  |  |
| * Physical activity is presented as a metabolic equivalent of task minutes per week. There are 9,973 missing data for male participants and 13,559 for female participants. | | | | | | | | | | |

**Table S8.** Number and proportion of incident cardiovascular disease events by handgrip strength after exact propensity score matching

| Male (n=53,588) | CVD events | *P* | Female (n=48,832) | CVD events | *P* |
| --- | --- | --- | --- | --- | --- |
| Non-MASLD (n=26,794) | 2,977 (11.1%) | <0.001 | Non-MASLD (n=24,416) | 1,947 (8.0%) | <0.001 |
| MASLD |  |  | MASLD |  |  |
| High HGS (n=8,202) | 1,020 (12.4%) |  | High HGS (n=7,929) | 694 (8.8%) |  |
| Middle HGS (n=10,738) | 1,448 (13.5%) |  | Middle HGS (n=9,490) | 1,012 (10.7%) |  |
| Low HGS (n=7,854) | 1,293 (16.5%) |  | Low HGS (n=6,997) | 904 (12.9%) |  |
| CVD, cardiovascular disease; MASLD, metabolic dysfunction-associated steatotic liver disease; HGS, handgrip strength | | | | | |

| **Table S9.** Cardiovascular disease risk according to handgrip strength in male and female participants after propensity score matching | | | | | | | | | |
| --- | --- | --- | --- | --- | --- | --- | --- | --- | --- |
| Male participants (n=53,588) | Events | Model 1 | | Model 2 | | Model 3 | | Model 4 | |
|  |  | HR | 95% CI | HR | 95% CI | HR | 95% CI | HR | 95% CI |
| Non-MASLD (n=26,794) | 2,977 | Reference | | Reference | | Reference | | Reference | |
| MASLD with high HGS (n=8,202) | 1,020 | 1.12 | (1.05,1.21) | 1.15 | (1.07,1.23) | 1.08 | (1.00,1.17) | 1.08 | (0.99,1.17) |
| MASLD with middle HGS (n=10,738) | 1,448 | 1.23 | (1.15,1.31) | 1.24 | (1.16,1.32) | 1.16 | (1.08,1.25) | 1.16 | (1.08,1.25) |
| MASLD with low HGS (n=7,854) | 1,293 | 1.53 | (1.44,1.64) | 1.48 | (1.39,1.59) | 1.39 | (1.29,1.51) | 1.38 | (1.28,1.49) |
| Female participants (n=48,832) | Events | Model 1 | | Model 2 | | Model 3 | | Model 4 | |
|  |  | HR | 95% CI | HR | 95% CI | HR | 95% CI | HR | 95% CI |
| Non-MASLD (n=24,416) | 1,947 | Reference | | Reference | | Reference | | Reference | |
| MASLD with high HGS (n=7,929) | 694 | 1.09 | (1.00,1.19) | 1.1 | (1.01,1.20) | 1.08 | (0.97,1.20) | 1.08 | (0.97,1.20) |
| MASLD with middle HGS (n=9,490) | 1,012 | 1.35 | (1.25,1.45) | 1.36 | (1.26,1.46) | 1.33 | (1.21,1.46) | 1.33 | (1.21,1.46) |
| MASLD with low HGS (n=6,997) | 904 | 1.66 | (1.53,1.80) | 1.62 | (1.50,1.75) | 1.58 | (1.43,1.74) | 1.57 | (1.43,1.73) |
| Model 1 was unadjusted. |  |  |  |  |  |  |  |  |  |
| Model 2 was adjusted for diabetes mellitus, hypertension, and dyslipidemia. | | | | |  |  |  |  |  |
| Model 3 was adjusted for body mass index, smoking, diabetes mellitus, hypertension, and dyslipidemia. | | | | | | | |  |  |
| Model 4 was adjusted for body mass index, smoking, diabetes mellitus, hypertension, dyslipidemia, and physical activity. | | | | | | | | | |
| MALSD, metabolic dysfunctiin-associated steatotic liver disease; HR, hazard ratios; CI, confidence intervals; HSG, handgrip strength | | | | | | | | | |

**Table S10.** Incident cardiovascular disease events according to handgrip strength among non-MASLD group

| Male (n=39,670) | CVD event | *P* | Female (n=86,395) | CVD event | *P* |
| --- | --- | --- | --- | --- | --- |
| High HGS (n=11,610) | 1,072 (9.2%) | <0.001 | High HGS (n=28,603) | 1,519 (5.3%) | <0.001 |
| Middle HGS (n=15,913) | 1,689 (10.6%) |  | Middle HGS (n=34,381) | 2,111 (6.1%) |  |
| Low HGS (n=12,147) | 1,568 (12.9%) |  | Low HGS (n=23,411) | 1,599 (6.8%) |  |
| CVD, cardiovascular disease; MALSD, metabolic dysfunctiin-associated steatotic liver disease; HGS, handgrip strength | | | | | |

**Table S11.** Cardiovascular disease risk according to handgrip strength in male and female participants in non-MASLD group

| Male subjects | Events | Model 1 | | Model 2 | | Model 3 | | Model 4 | |
| --- | --- | --- | --- | --- | --- | --- | --- | --- | --- |
|  |  | HR | 95% CI | HR | 95% CI | HR | 95% CI | HR | 95% CI |
| High HGS (n=11,610) | 1,072 | Reference | | Reference | | Reference | | Reference | |
| Middle HGS (n=15,913) | 1,689 | 1.16 | (1.08,1.25) | 1.13 | (1.05,1.23) | 1.14 | (1.05,1.23) | 1.14 | (1.05,1.23) |
| Low HGS (n=12,147) | 1,568 | 1.43 | (1.33,1.55) | 1.35 | (1.25,1.46) | 1.35 | (1.24,1.46) | 1.35 | (1.24,1.45) |
| Female subjects | Events | Model 1 | | Model 2 | | Model 3 | | Model 4 | |
|  |  | HR | 95% CI | HR | 95% CI | HR | 95% CI | HR | 95% CI |
| High HGS (n=28,603) | 1,519 | Reference | | Reference | | Reference | | Reference | |
| Middle HGS (n=34,381) | 2,111 | 1.17 | (1.09,1.25) | 1.16 | (1.08,1.23) | 1.16 | (1.08,1.24) | 1.15 | (1.08,1.23) |
| Low HGS (n=23,411) | 1,599 | 1.30 | (1.21,1.40) | 1.27 | (1.18,1.36) | 1.26 | (1.17,1.35) | 1.25 | (1.17,1.34) |
| Model 1 was unadjusted. |  |  |  |  |  |  |  |  |  |
| Model 2 was adjusted for diabetes mellitus, hypertension, and dyslipidemia. | | | | |  |  |  |  |  |
| Model 3 was adjusted for body mass index, smoking, diabetes mellitus, hypertension, and dyslipidemia. | | | | | | | |  |  |
| Model 4 was adjusted for body mass index, smoking, diabetes mellitus, hypertension, dyslipidemia, and physical activity. | | | | | | | | | |
| MALSD, metabolic dysfunctiin-associated steatotic liver disease; HR, hazard ratios; CI, confidence intervals; HSG, handgrip strength | | | | | | | | | |

**Table S12.** Cardiovascular disease risk according to handgrip strength in male and female participants in non-MASLD and MASLD group

| Male subjects | Events | Model 1 | | Model 2 | | Model 3 | | Model 4 | |
| --- | --- | --- | --- | --- | --- | --- | --- | --- | --- |
|  |  | HR | 95% CI | HR | 95% CI | HR | 95% CI | HR | 95% CI |
| Non-MASLD with high HGS (n= 11,610) | 1,072 | Reference | | Reference | | Reference | | Reference | |
| Non-MASLD with middle HGS (n= 15,913) | 1,689 | 1.16 | (1.08-1.25) | 1.14 | (1.06-1.23) | 1.14 | (1.06-1.24) | 1.14 | (1.06-1.23) |
| Non-MASLD with low HGS (n= 12,147) | 1,568 | 1.43 | (1.33-1.55) | 1.36 | (1.26-1.47) | 1.37 | (1.26-1.48) | 1.36 | (1.26-1.47) |
| MASLD with high HGS (n= 12,640) | 1,675 | 1.47 | (1.36-1.59) | 1.28 | (1.18-1.38) | 1.18 | (1.09-1.29) | 1.18 | (1.09-1.28) |
| MASLD with middle HGS (n=16,815) | 2,489 | 1.66 | (1.54-1.78) | 1.41 | (1.31-1.52) | 1.31 | (1.21-1.42) | 1.31 | (1.21-1.41) |
| MASLD with low HGS (n=12,790) | 2,353 | 2.11 | (1.97-2.27) | 1.72 | (1.59-1.85) | 1.59 | (1.47-1.72) | 1.58 | (1.46-1.71) |
| Female subjects | Events | Model 1 | | Model 2 | | Model 3 | | Model 4 | |
|  |  | HR | 95% CI | HR | 95% CI | HR | 95% CI | HR | 95% CI |
| Non-MASLD with high HGS (n= 28,603) | 1,519 | Reference | | Reference | | Reference | | Reference | |
| Non-MASLD with middle HGS (n= 34,381) | 2,111 | 1.17 | (1.09-1.24) | 1.16 | (1.08-1.24) | 1.16 | (1.08-1.23) | 1.15 | (1.08-1.23) |
| Non-MASLD with low HGS (n= 23,411) | 1,599 | 1.30 | (1.21-1.40) | 1.27 | (1.19-1.37) | 1.26 | (1.18-1.35) | 1.25 | (1.17-1.34) |
| MASLD with high HGS (n= 10,763) | 987 | 1.74 | (1.61-1.89) | 1.37 | (1.27-1.49) | 1.26 | (1.15-1.38) | 1.25 | (1.14-1.38) |
| MASLD with middle HGS (n=12,813) | 1,377 | 2.07 | (1.92-2.23) | 1.62 | (1.50-1.74) | 1.49 | (1.37-1.62) | 1.48 | (1.36-1.61) |
| MASLD with low HGS (n=9,677) | 1,287 | 2.61 | (2.42-2.81) | 1.95 | (1.81-2.11) | 1.79 | (1.64-1.95) | 1.77 | (1.62-1.93) |
| Model 1 was unadjusted. |  |  |  |  |  |  |  |  |  |
| Model 2 was adjusted for diabetes mellitus, hypertension, and dyslipidemia. | | | | |  |  |  |  |  |
| Model 3 was adjusted for body mass index, smoking, diabetes mellitus, hypertension, and dyslipidemia. | | | | | | | |  |  |
| Model 4 was adjusted for body mass index, smoking, diabetes mellitus, hypertension, dyslipidemia, and physical activity. | | | | | | | | | |
| MALSD, metabolic dysfunctiin-associated steatotic liver disease; HR, hazard ratios; CI, confidence intervals; HSG, handgrip strength | | | | | | | | | |

**(a)**


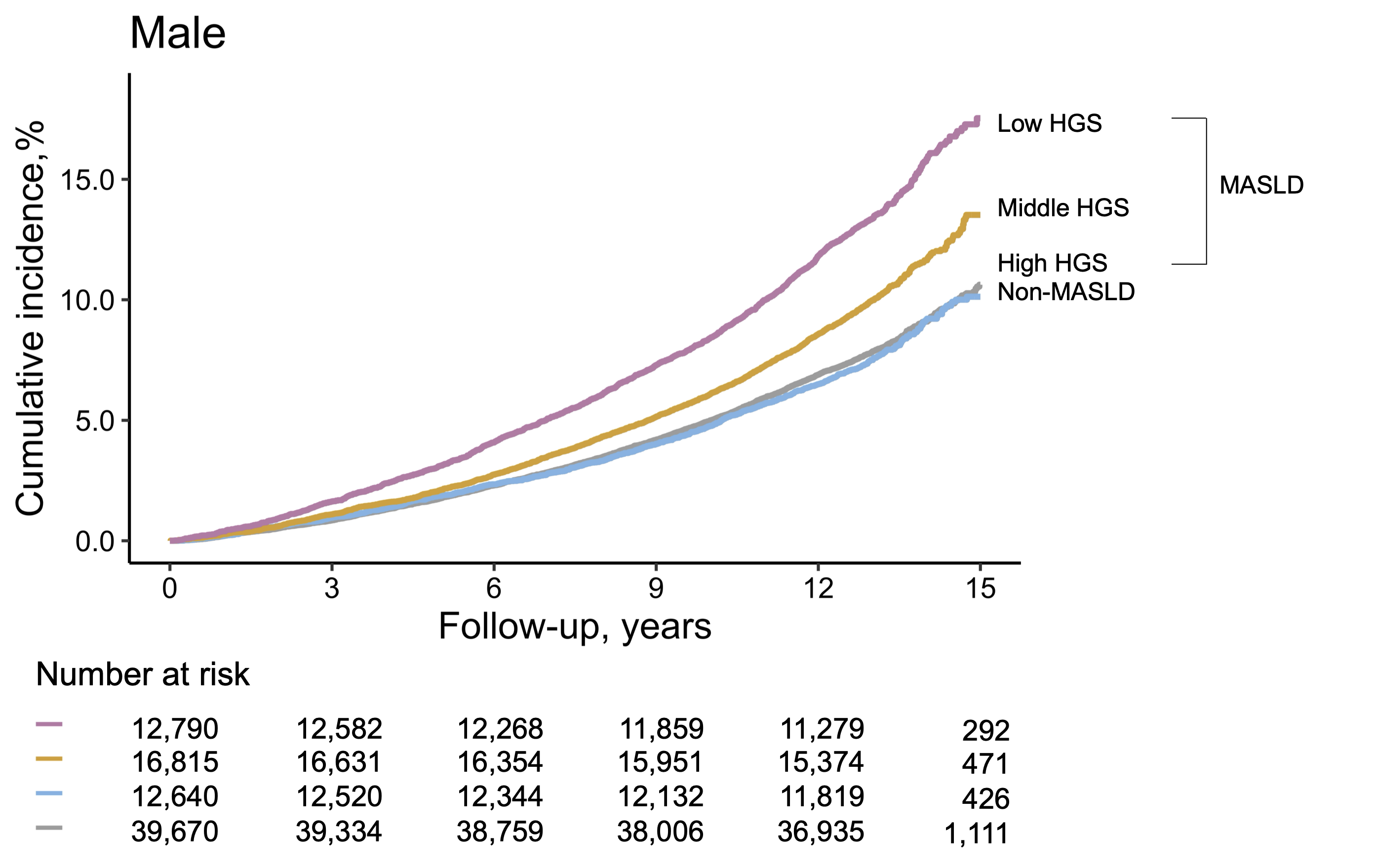


**(b)**


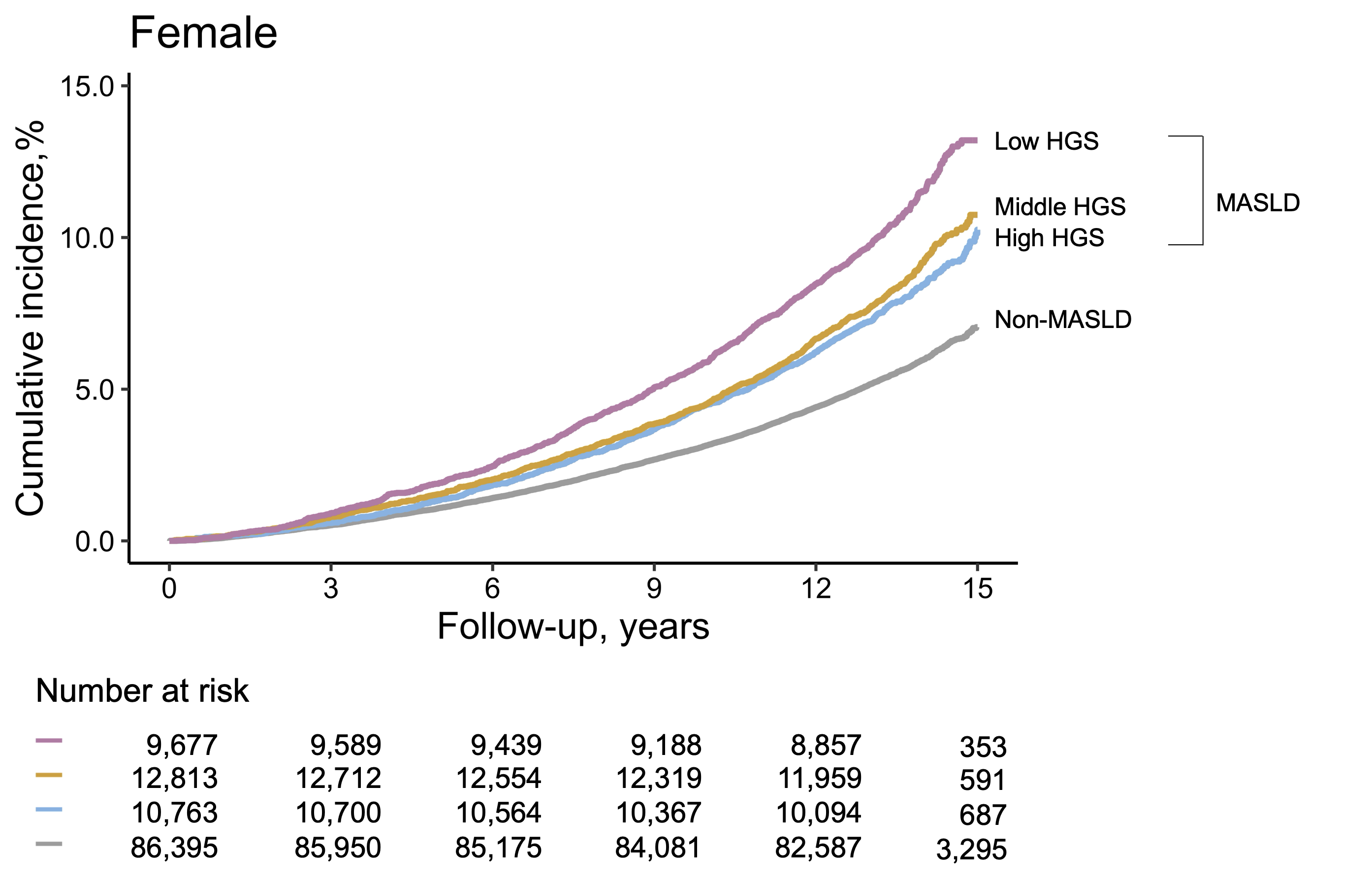


**Figure S1.** Cumulative incidence of mortality events according to handgrip strength in male (a) and female (b) subjects.

**(a)**

**
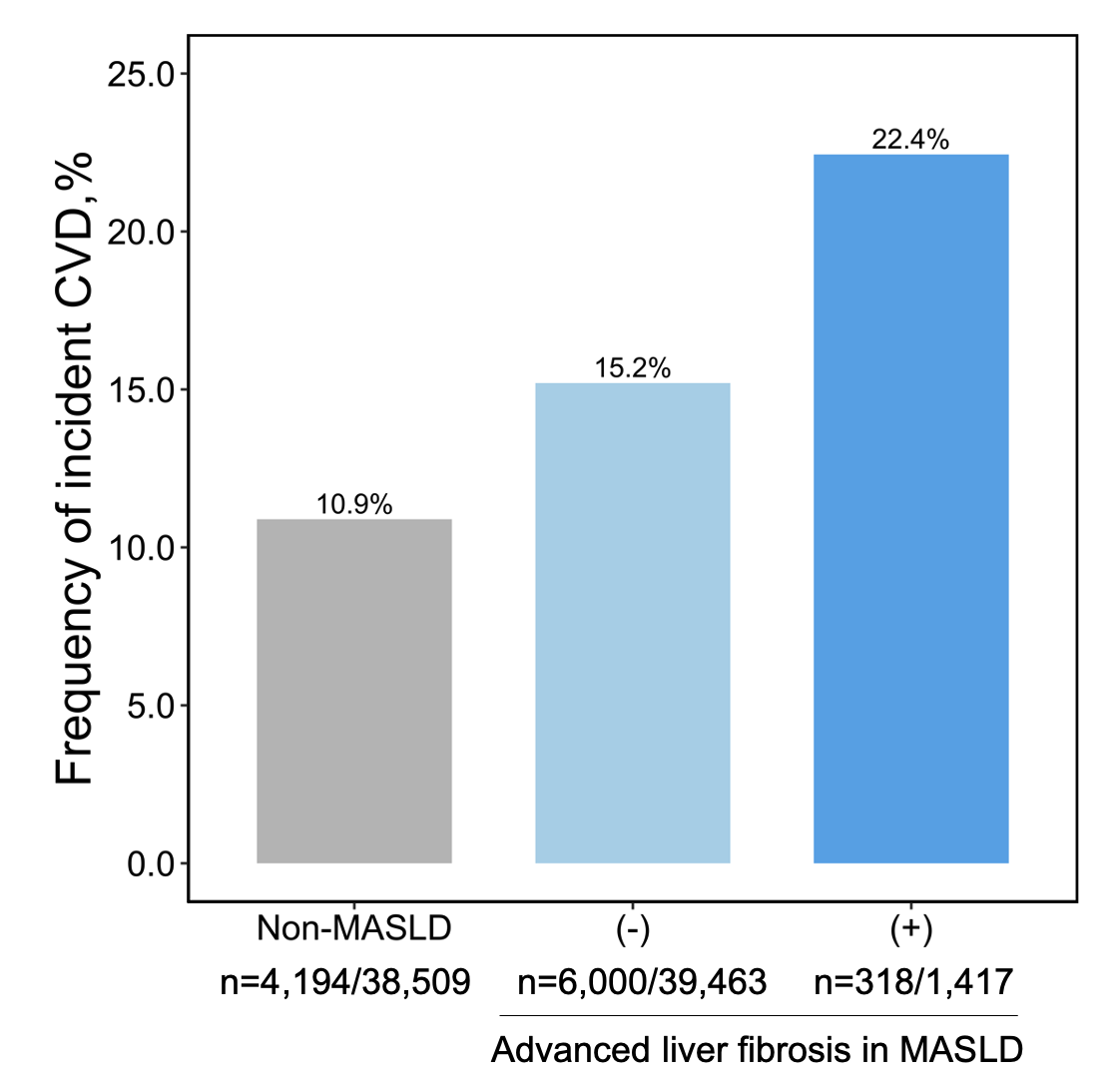
**

**(b)**

**
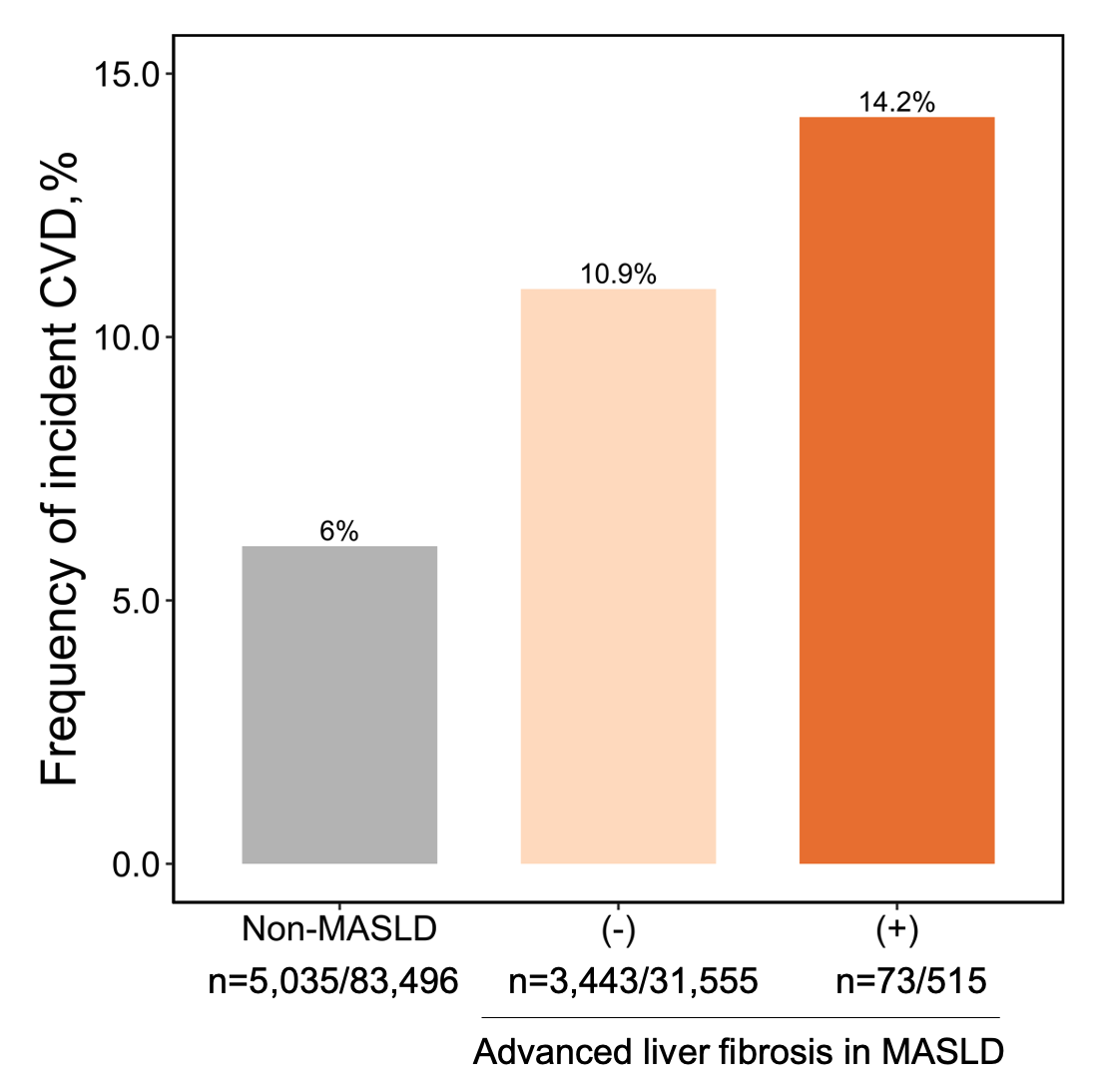
**

**Figure S2.** Incidence of CVD events in non-MASLD participants and in MASLD participants with or without advanced liver fibrosis, for males (a) and females (b). CVD, cardiovascular disease; MASLD, metabolic dysfunction-associated steatotic liver disease

**(a)**

**
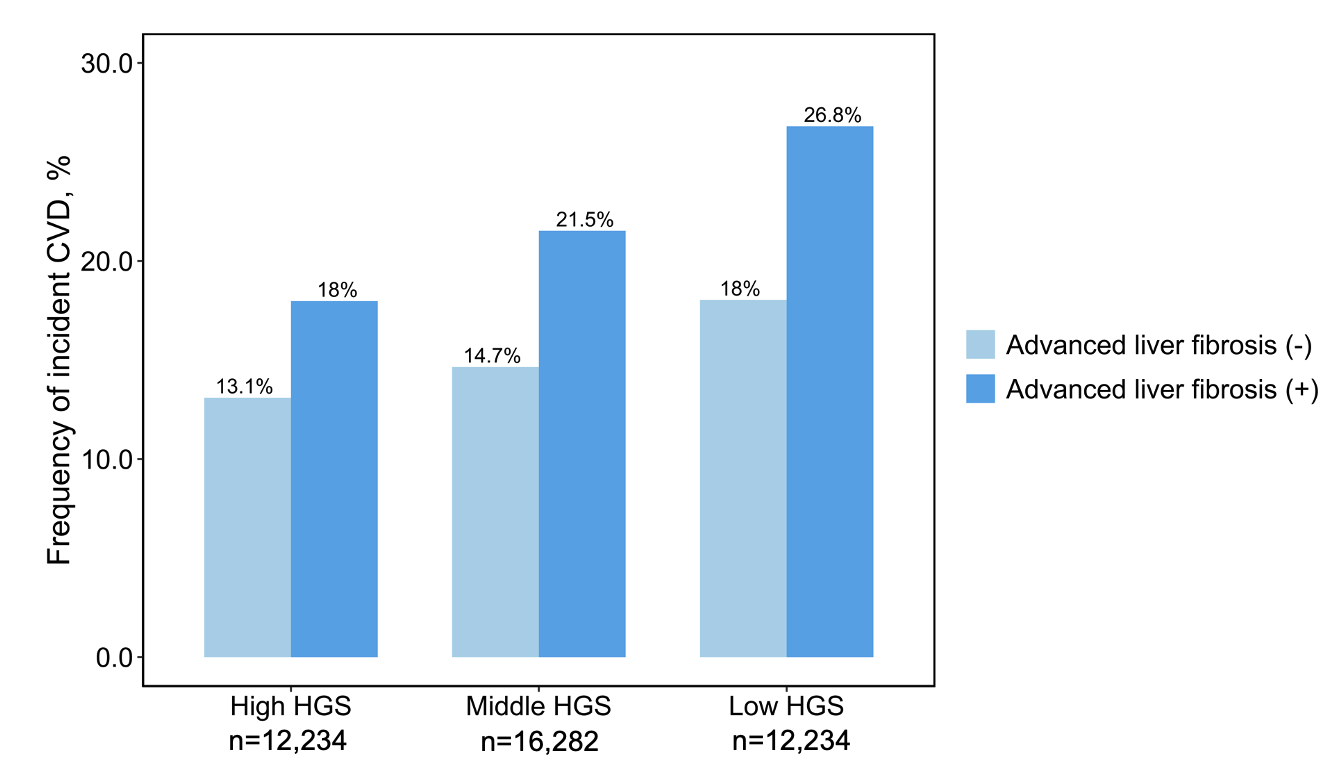
**

**(b)**

**
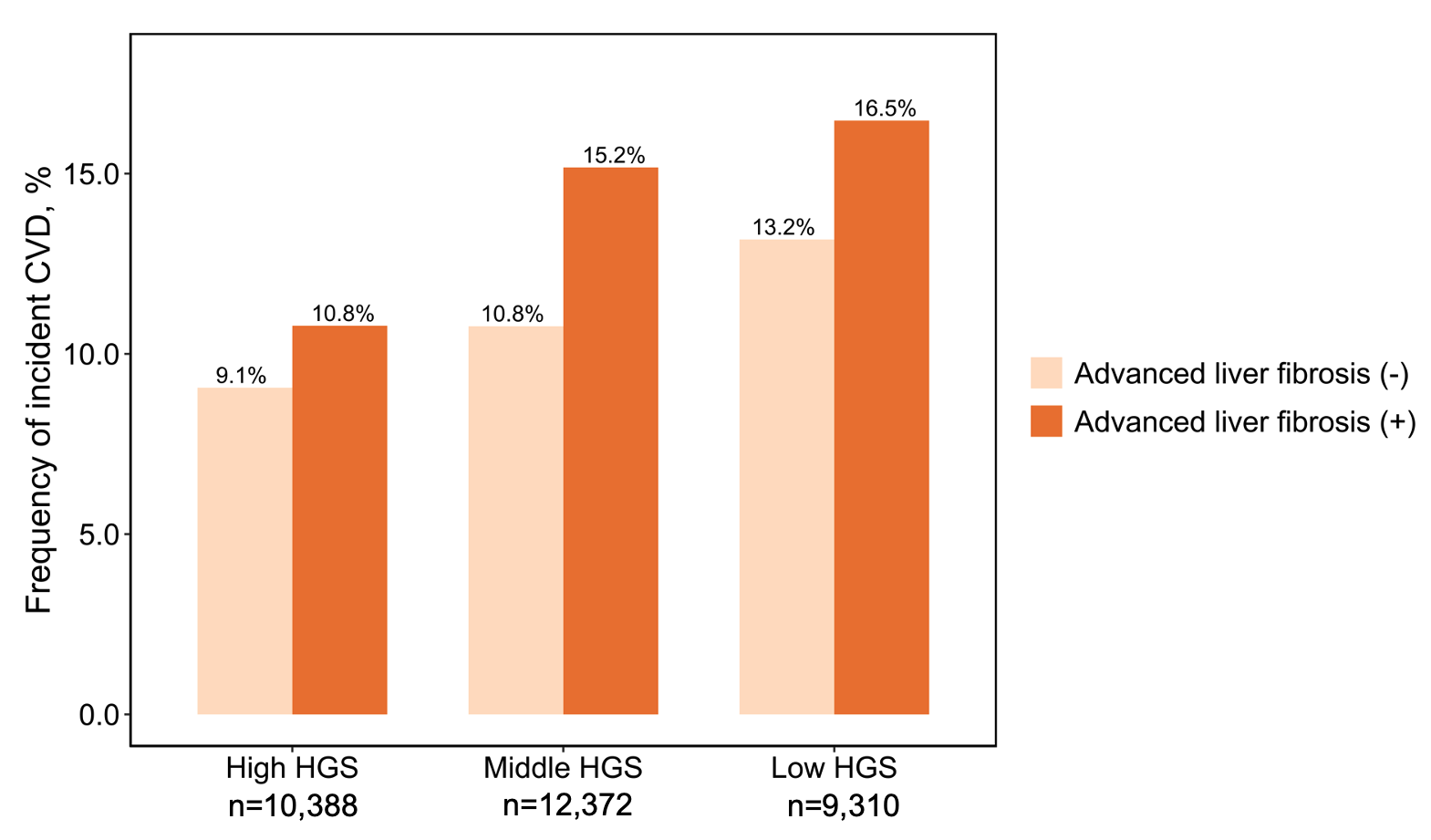
**

**Figure S3.** Incidence of CVD events by the presence or absence of advanced liver fibrosis within each HGS group for males (A) and females (B) in MASLD. CVD, cardiovascular disease; MASLD, metabolic dysfunction-associated steatotic liver disease; HGS, handgrip strength
